# Investigating the Secondary Use of Clinical Research Data: Protocol for a Mixed Methods Study

**DOI:** 10.2196/44875

**Published:** 2023-03-06

**Authors:** Naomi Waithira, Evelyne Kestelyn, Keitcheya Chotthanawathit, Anne Osterrieder, Mavuto Mukaka, Trudie Lang, Phaik Yeong Cheah

**Affiliations:** 1 Centre for Tropical Medicine and Global Health Nuffield Department of Medicine University of Oxford Oxford United Kingdom; 2 Mahidol-Oxford Tropical Medicine Research Unit Bangkok Thailand; 3 Oxford University Clinical Research Unit Ho Chi Minh City Vietnam

**Keywords:** data reuse, data sharing, secondary data use, clinical trials data, artificial intelligence, machine learning, individual patient data, clinical research, barriers, online survey, mixed methods, low- and middle-income country

## Abstract

**Background:**

The increasing emphasis to share patient data from clinical research has resulted in substantial investments in data repositories and infrastructure. However, it is unclear how shared data are used and whether anticipated benefits are being realized.

**Objective:**

The purpose of our study is to examine the current utilization of shared clinical research data sets and assess the effects on both scientific research and public health outcomes. Additionally, the study seeks to identify the factors that hinder or facilitate the ethical and efficient use of existing data based on the perspectives of data users.

**Methods:**

The study will utilize a mixed methods design, incorporating a cross-sectional survey and in-depth interviews. The survey will involve at least 400 clinical researchers, while the in-depth interviews will include 20 to 40 participants who have utilized data from repositories or institutional data access committees. The survey will target a global sample, while the in-depth interviews will focus on individuals who have used data collected from low- and middle-income countries. Quantitative data will be summarized by using descriptive statistics, while multivariable analyses will be used to assess the relationships between variables. Qualitative data will be analyzed through thematic analysis, and the findings will be reported in accordance with the COREQ (Consolidated Criteria for Reporting Qualitative Research) guidelines. The study received ethical approval from the Oxford Tropical Research Ethics Committee in 2020 (reference number: 568-20).

**Results:**

The results of the analysis, including both quantitative data and qualitative data, will be available in 2023.

**Conclusions:**

The outcomes of our study will offer crucial understanding into the current status of data reuse in clinical research, serving as a basis for guiding future endeavors to enhance the utilization of shared data for the betterment of public health outcomes and for scientific progress.

**Trial Registration:**

Thai Clinical Trials Registry TCTR20210301006; https://tinyurl.com/2p9atzhr

**International Registered Report Identifier (IRRID):**

DERR1-10.2196/44875

## Introduction

### The Value of Clinical Research Data

Although clinical research data are generated to answer specific questions, the data can be used for purposes other than those of the original planned analyses to increase medical understanding and improve the general health of the population [1,2].

Many recent advances in medicine are credited to the reuse of existing research data. During the outbreaks of H1N1; MERS (Middle East Respiratory Syndrome); Ebola; Zika; and, more recently, COVID-19, decision-making authorities such as the World Health Organization depended on the analysis of historical epidemiological and clinical data to understand disease progression patterns and associated risk factors. This knowledge was the basis of recommendations for nonpharmaceutical interventions to control the spread of the diseases [3-6]. Guidelines for treatment therapies and preventive medicines depend on findings from clinical trials. The design and interpretation of these clinical trials rely heavily on data from previous studies in, for instance, the calculation of sample sizes, definition of outcomes, selection of interventions and control treatments, and establishment of follow-up duration [7-10]. Existing clinical research data have also been used to answer completely new research questions for teaching purposes and to reproduce results of published research [11].

As the volume of research data continues to rise, there is greater potential for faster and innovative discoveries in diagnostics and the treatment and prevention of diseases through traditional analyses and machine learning technologies [12].

Recognizing the value of data reuse, funders, journals, and research bodies are establishing policies and making substantial resource investments in infrastructure to facilitate data sharing [13,14]. In 2003, the National Institutes of Health introduced the first funder-driven data sharing policy. By 2016, key health research funders required individual participant-level data that support findings of clinical trials to be made accessible at the time of the publication of study results [15-17]. Journals and publishers followed suit, with the majority of them mandating the inclusion of data availability statements in articles for publication [18]. These statements describe how and when data can be accessed and on which repository data are stored. Numerous repositories have been established by funders, pharmaceutical companies, academic research institutions, governments, and discipline-specific consortia [19]. In 2016, at the G20 Hangzhou Summit, the FAIR (Findable, Accessible, Interoperable, and Reusable) principles were endorsed. The FAIR principles aim to optimize the reuse of data sets by ensuring that data are findable; are accessible; are presented in a standardized, interoperable format; and exist on terms that allow reuse [20,21]. Groups such as the Clinical Data Interchange Standards Consortium have defined standard ways of describing data and metadata [22]. By standardizing data and metadata, researchers and algorithms can correctly interpret data without the need for intermediary translation or curation. Analytical tools, such as TwoRavens [23], now allow users to run statistical models and view summary statistics on data held in repositories. The implementation of this ecosystem requires staff with specialized skills; research institutions are investing in hiring and training data managers, programmers, and data scientists to develop interfaces and curate data and metadata.

It is expected that these investments in infrastructure will increase the usage of existing data and, consequently, generate a rich reference for evidence-based decision-making.

### Rationale

Despite the efforts to facilitate data sharing, it is still unclear if and how shared data sets are used and whether the anticipated benefits are being realized. Previous studies described data usage trends based on data sets held in repositories and those from studies that are registered in clinical trial registries [24]. Negative results and studies terminated prematurely are often not published in repositories, yet data from these studies may hold useful insights for planning future research [25]. A substantial proportion of studies conducted in low- and middle-income countries (LMICs), especially noninterventional studies, are not registered [26]. Data sets generated in LMICs are particularly valuable, given the high disease burden in LMICs and the low volume of research being conducted in these areas [27-30]. To our knowledge, no recently published work has examined current barriers to and opportunities for maximizing data utility in LMICs.

### Objectives

Against this background, our study aims to bridge the evidence gap by (1) characterizing how clinical research data sets are reused; (2) describing what impact, if any, data reuse has had on scientific research and general public health; and (3) defining barriers to and opportunities for promoting the ethical, efficient, and equitable reuse of data based on the perspectives of secondary data users.

As previous studies primarily focused on participants from high-income countries, we will target individuals working with data collected from LMICs for the interviews and aim to include respondents from both high-income countries and LMICs in the survey.

## Methods

### Overview

We will conduct a mixed methods study comprising an anonymous web-based survey and semistructured in-depth interviews. The survey will provide data from a large population, while the interviews will generate data on the detailed and contextualized perspectives of individual data users. We aim to include a wide spectrum of participants to reflect the diversity in research areas, career levels, and geographical locations.

### Recruitment

#### Cross-sectional Survey

We will include at least 400 participants, of whom half will be individuals with a history of data reuse (data users), and the other half will be participants with no history of data reuse (nonusers). For the data user group, we will investigate what kinds of data sets were used, how the data were accessed, the purposes for requesting the data, and outcomes of the secondary analyses. We will additionally probe for information on challenges faced in accessing and using the data. For the nonuser group, we will determine reasons for not using shared data sets and identify perceptions on data reuse.

The inclusion criteria are as follows: (1) individuals aged ≥18 years, (2) researchers and professionals working in clinical research or with clinical research data, and (3) those who provide written consent to participate in the study. Individuals with no access to a computer, a mobile device, or the internet will be excluded, as data collection will be mainly conducted via the internet.

Potential participants will be requested to provide consent by ticking a checkbox to confirm that they agree to participate in the study. Respondents who do not provide consent will not be able to access the survey.

Data users will be directed to a page for data users, while nonusers will be directed to a page for nonusers. It will take 3 to 7 minutes to complete the survey for data users and 1 to 2 minutes to complete the survey for nonusers.

The survey will be designed based on existing literature. A panel of experts will be involved in the survey design, including social scientists and ethicists (for informing how questions are framed), a statistician (for the sampling strategy and the development of measurement scales and scoring schemes), a data manager (for survey development and data extractions), and a group of researchers and data users (for piloting). We will use the cognitive debriefing method to pilot the survey among respondents who are similar to the target population. The pilot testing group will be selected purposively for practical reasons and will include individuals from diverse research areas, career levels, and geographical locations. During the pilot, we will check the comprehension of each question; the ability to recall answers; and whether questions are appropriate, are clear, and have sufficient response options. Feedback from the pilot phase will be incorporated in the final survey before deployment for data collection. To achieve demographic diversity, the survey will be translated into other languages, such as Spanish, French, and Portuguese. We will translate the survey by following the TRAPD (Translation, Review, Adjudication, Pretesting, and Documentation) model [31]. Professional translators will perform the initial translations before a review by bilingual individuals. To guarantee that the translated survey accurately represents the intended meaning, it will be pretested with native speakers who are comparable to the target audience.

The survey instrument focuses on the following four main areas: respondents’ demographics, the nature of data use, challenges with data use, and interventions to enhance data use (Multimedia Appendices 1-5). Conditional logic will be applied to display nested questions that are dependent on the respondents’ previous answers. For example, respondents who have published results that are based on secondary analyses will be asked to indicate the number of publications generated from those analyses. Similarly, questions on the history of data use will not be displayed for nonusers.

To reach the target population, the survey will be advertised on forums for researchers and secondary data users, such as the Global Health Network and the COVID-19 Clinical Research Coalition; in conferences; and through the collaborating institutions’ social media platforms. Participants will also be encouraged to share the survey with their colleagues. The survey will be conducted globally. The data will be reviewed after the number of recruited participants reaches one-third (132/400, 33%) of the target sample size to monitor the distribution of respondents by geographical location, research area, and occupation. The results of the review will be used to strategize the advertisement and distribution of the survey to target specific groups.

#### Interviews

Semistructured in-depth interviews will be conducted with 20 to 40 data users or until a state of data saturation is reached. Data saturation occurs when further interviews no longer produce new insights or themes [32]. Considering limitations on travel and on holding meetings resulting from the COVID-19 pandemic, we will primarily use web-based or remote methods for data collection. In-person interviews may be conducted when it is safe to do so, in compliance with local regulations.

The inclusion criteria are as follows: (1) individuals aged ≥18 years, (2) researchers or professionals using clinical research data shared by other researchers for secondary purposes, and (3) those who provide consent to participate in the study.

Potential participants will be identified through a web-based search for publications that are based on secondary analyses, focusing on researchers and professionals who have used data from LMICs. The corresponding authors will be contacted by email and invited to participate in interviews. To reach data users who have not yet generated publications, we will invite individuals who have requested data sets from institutional data repositories. Individuals who are interested in participating in the study will be sent the participant information sheet and consent form by email. The information sheet will describe the nature of the study, what the interviews will entail, and details on how data will be stored and processed during and after the study. It will be clearly stated that the participants are free to withdraw from the study at any time, for any reason, and with no obligation to give the reason for withdrawal. The participants will be allowed as much time as they wish to consider the information, and they will be given the opportunity to raise questions with the investigator or other independent parties to decide whether they will participate in the study. Interviews will then be scheduled at mutually convenient times. Audio-recorded consent will be obtained prior to asking the interview questions. The interviewer will document that verbal consent was obtained on a consent form. The investigator will keep a copy of the signed form in the study file and send a copy to the participants by email. For interviews that are held in person, the participants will sign the consent form prior to start of the interview. The interviews are estimated to last 40 to 60 minutes. Multimedia Appendix 6 shows the interview guide.

It is likely that participants of the in-depth interviews may be included in the survey. However, as the survey is anonymous, it will not be possible to identify individuals who participate in both the survey and the interviews.

### Data Management

Quantitative data will be collected on the Jisc Online Surveys platform, which is managed through the University of Oxford [33]. Data will be exported from Jisc in CSV format for analysis and long-term preservation. The survey database will be retained for 6 months after the completion of data collection, after which all data will be exported from the survey platform and stored indefinitely in access-controlled servers at Mahidol Oxford Tropical Medicine Research Unit (MORU)—a collaboration between the University of Oxford and Mahidol University that carries out clinical and public health research [34]. Deidentified data may be uploaded on data repositories or shared with other researchers, in line with the data sharing policies of MORU and collaborating institutions, as applicable.

Qualitative data will include detailed summary notes and audio recordings of the interviews. Web-based interviews will be conducted in English via Microsoft Teams (Microsoft Corporation)—a secure access-restricted platform. Transcripts of the interviews will be generated by using the Microsoft Teams transcription feature. The draft transcripts will be manually checked for accuracy line by line. Using the Health Insurance Portability and Accountability Act safe harbor method, direct and indirect identifiers, such as references to names of individuals or institutions, will be removed from the transcripts [35]. The deidentified transcripts will be uploaded to the latest version of NVivo (QSR International) software for storage and organization. Audio recordings and email correspondence will be stored separately from deidentified transcripts to preserve the confidentiality and privacy of participants. After the completion of analyses and the reporting of study results, audio recordings and original transcripts will be deleted. Deidentified transcripts, summary notes, and coded data will be stored indefinitely in access-controlled servers at MORU.

### Statistical Analysis

#### Sampling

Participants will be enrolled through nonprobability sampling, as no suitable sample frame exists for the population being studied. A minimum of 200 participants is adequate for estimating any prevalence of a response, assuming a 50% prevalence rate, with 95% confidence and a precision of around 7%. Considering a minimum of 200 data users and 200 nonusers, the minimum total sample size for the web-based survey is 400 participants. A sample size higher than 400 participants will increase the precision of the prevalence estimates.

The sample size for the qualitative study will depend on when data saturation is reached. We estimate that a purposive sample of between 20 and 40 participants will be adequate, following the rule of thumb for the estimation of sample sizes for in-depth interviews in mixed methods studies [36].

#### Analysis

Quantitative survey data will be analyzed by using Stata 15.0 (or later; StataCorp LLC) software. Frequency counts and percentages will be used to summarize categorical data. Associations between categorical variables will be assessed by using Pearson chi-square tests or Fisher exact tests, as appropriate. Data will be presented in tables, graphical displays, and summary statistics. Further analyses for determining the significance of relationships between variables will be performed when necessary. Tests of significance will be performed at the 5% significance level (α=.05) for quantitative data.

Qualitative data will be synthesized by using thematic analysis [37], and the findings will be reported in accordance with the COREQ (Consolidated Criteria for Reporting Qualitative Research) guidelines. Open coding will be performed by breaking data into discrete parts and assigning them codes. Through axial coding, related codes will be combined to form subthemes. Related subthemes will then be collated to form themes, and the relationships between themes will be presented using thematic maps. The themes will be described in detail within the study report, including verbatim quotes from participants as illustrations.

To ensure the validity and trustworthiness of the study results, data will be coded by 2 independent individuals. For codes that are contradictory, divergences will be outlined and discussed in the report.

### Ethical Considerations

Ethical approval was obtained in December 2020, prior to initiating the study, from the University of Oxford’s Tropical Research Ethics Committee (reference number: 568-20), which will provide overall oversight of the study. Institutional review board approvals may be obtained for collaborating institutions, as applicable.

Our study will pose minimal risk and harm to participants. Although there are no immediate benefits for study participants, participation in the study will afford them an opportunity to contribute to the generation of new knowledge that will potentially increase the reuse of data for public good.

The main ethical risks relate to privacy and confidentiality, particularly in the in-depth interviews. Care will be taken to maintain privacy during interviews and interactions with participants. Data containing person-identifying information will be stored securely and confidentially. Study documents and data will be accessible to study staff and authorized personnel only. The web-based survey is completely anonymous. Names, email addresses, IP addresses, or other person-identifying details will not be collected in the survey.

The study will comply with the European Union General Data Protection Regulation, as described in the participant information sheets for the survey (Multimedia Appendices 1-5) and the interviews (Multimedia Appendix 7). Personal data will be stored until the final analyses are completed, while anonymized data will be stored indefinitely.

### Data Sharing

With participants’ consent, anonymized data will be uploaded on data repositories and shared with other researchers, in line with the collaborating institutions’ data sharing policies. We will share the information we collect in ways that do not reveal individual participants’ identities.

### Informed Consent

Participation in the study is voluntary. All participants must provide informed consent before involvement in the survey or in the interviews. Survey participants will only be able to access survey questions after providing consent. For in-depth interviews, verbal consent will be obtained at the start of the interview.

### Dissemination

The results of our study will be primarily shared through the publication of results in peer-reviewed journals and through scientific presentations in webinars and seminars.

## Results

The results of the analysis, including both quantitative data and qualitative data, will be available in 2023.

## Discussion

### Overview

Data sharing is widely regarded as a positive development for scientific and technological advancement. By making research data available beyond the primary research team, the data can be scrutinized, reused, and built upon, leading to greater insights, innovation, and collaboration. Research indicates that data sharing is becoming increasingly mandated, with funding agencies, academic institutions, and journals requiring researchers to make their data available. This is due to the belief that data sharing can lead to more reproducible and trustworthy results and an increase in the visibility of researchers in the scientific community. Despite these mandates, it is important to note that the uptake of data sharing practices among researchers is still relatively low. Although data sharing can bring significant benefits to scientific advancement and collaboration, it also requires significant investments in terms of time, resources, and technology. It remains unclear whether the benefits of data sharing are actually worth the costs. Furthermore, data sharing can also amplify challenges, such as the need for proper data curation and privacy protection. To date, there is sparse literature on the barriers and drivers of clinical research data reuse based on the perspectives of secondary data users [38-43]. Although some secondary data users cite the lack of access to quality and relevant data as a challenge, ironically, other researchers suggest that the clinical research data sets that exist in the public domain are grossly underutilized [1,44].

### Expected Findings

Through our exploratory study, we aim to gain insights into how shared data sets are used, analyze the impact of secondary usage, and document barriers and facilitators of secondary data use based on the perspectives of data users.

### Limitations

The survey participants will be selected by using nonrandom sampling, which means that the results may not accurately reflect the characteristics of the whole population and may be affected by bias in the selection process. Additionally, it is possible that the survey may only reach those who regularly use the web-based platforms where the survey is promoted, potentially resulting in an incomplete representation of the target population. Although it is acknowledged that the survey results may not be generalizable to a larger population, the data from our exploratory study will provide ideas and hypotheses that could guide future research. Additionally, the in-depth interviews are expected to provide valuable and contextualized information.

The study is limited to the description of the current state of data reuse, and it will not explore causal relationships. However, the findings from our study can be used as a foundation to design future studies that establish cause-and-effect relationships in secondary data use and explore perspectives of nonusers.

### Conclusions

In conclusion, the findings from our work could offer insights into informing strategies for increasing the utilization of existing clinical research data sets in a manner that benefits researchers and populations, particularly in LMICs.

## Appendix

Multimedia Appendix 1 Survey instrument in English.

Multimedia Appendix 2 Survey instrument in Spanish.

Multimedia Appendix 3 Survey instrument in French.

Multimedia Appendix 4 Survey instrument in Portuguese.

Multimedia Appendix 5 Survey instrument in Vietnamese.

Multimedia Appendix 6 Interview guide.

Multimedia Appendix 7 Participant information sheet: In-depth interviews.

## Abbreviations

COREQ: Consolidated Criteria for Reporting Qualitative Research
FAIR: Findable, Accessible, Interoperable, and Reusable
LMIC: low- and middle-income country
MERS: Middle East Respiratory Syndrome
MORU: Mahidol Oxford Tropical Medicine Research Unit
TRAPD: Translation, Review, Adjudication, Pretesting, and Documentation

## Data Availability

The data sets generated and analyzed during the study will be available from the Mahidol Oxford Research Tropical Medicine Research Unit Data Access Committee on reasonable request [45].
