# Supplementary material for: Investigating the Secondary Use of Clinical Research Data: Protocol for a Mixed Methods Study
Source: JMIR Res Protoc. 2023 Mar 6;12:e44875. doi: 10.2196/44875 (PMC10028503; doi:10.2196/44875)
Supplement: Multimedia Appendix 1 [file resprot_v12i1e44875_app1.pdf]

This survey is part of a study led by researchers from University of Oxford to understand if and how medical researchers use data collected by others. Even if you do not use data collected by others, your insights are useful for this study. Your responses are anonymous. The survey takes **3 -7** minutes to complete.

**What is the purpose of the research?**
It is increasingly becoming the norm to share data from clinical research studies. We seek to understand if and how shared datasets are reused, what challenges exist in accessing and reusing the data and what impact data reuse has had on scientific research and general public health.

**What are the possible benefits of this research?**
Data from this study will help to define what measures should be implemented to increase effective secondary data use. We anticipate that increased reuse of data will result in improved quality and transparency in science, improved public health and patient outcomes, and better return on investment in research.

**Study team and information**
The study is led by researchers from University of Oxford and Mahidol Oxford Tropical Medicine Research Unit with collaborators in UK, Kenya and Vietnam. The study has been approved by the Oxford Tropical Research Ethics Committee (OxTREC) reference number: 568-20.

**Data protection**
In the course of completing the survey, you will have provided information about yourself ('personal data'). Data protection regulation requires that we state the legal basis for processing information about you. In the case of research, this is 'a task in the public interest.' The University of Oxford is the data controller and is responsible for looking after your information and using it in accordance with the General Data Protection Regulation and associated data protection legislation. Your data will be held securely in accordance with the University's policies and procedures. Further information is available on the University's Information Security website. Information on your rights in relation to your personal data are explained here.

The findings from this research project will be disseminated through research reports, publications/articles and presentations. We are processing your data for these purposes only because you have given us your consent to do so, by ticking the appropriate box. Your responses will be anonymous, as the survey does not collect your name, email address or IP address. Because of this, we will not be able to withdraw your responses retrospectively, after you have submitted them. If you exit the survey before submitting your responses, your data will not be stored.

**Contact**
If you wish to raise any queries or concerns about our use of your data, please contact us at *reuse@tropmedres.ac* or on Telephone +66 02 203 6333 Ext 8302
If you wish to contact someone independent of the study team, you can email the Oxford Tropical Research Ethics Committee (OxTREC) by email: oxtrec@admin.ox.ac.uk.

☐ I agree to take part in the survey. I understand that my participation is voluntary and that I am free to withdraw at any time (please tick).

**Have you used clinical research data that has been shared by other researchers?**

- ◯ Yes
- ◯ No

**1) What types of data, shared by other researchers, have you used?** *(tick all that apply)*

- ☐ Clinical trials data
- ☐ Cross-sectional health surveys
- ☐ Observational cohort data
- ☐ Surveillance data
- ☐ Administrative data
- ☐ Patient / Disease registries
- ☐ Pharmacology data
- ☐ Molecular biology data
- ☐ Omics data (Genomics, Transcriptomics, Proteomics, Epigenomics, Metabolomics)
- ☐ Social science data
- ☐ Health economics data
- ☐ Qualitative data
- ☐ Other

You selected Qualitative or 'Other' data. Please specify what kind of research generated the data

**2) How did you access the datasets?** *(tick all that apply)*

- ☐ Request to data collector/custodian
- ☐ Download from a public website/repository
- ☐ Request through a Data Access Committee
- ☐ Other

You selected Other, please specify:

**3) How many times have you requested data in the last 5 years ?** (*if you cannot remember the exact number of requests,you may provide an estimate*)

In 2017

In 2018

In 2019

In 2020

In 2021

**4) What did you use the data for?** *(Tick all that apply)*

- ☐ To plan and design a new study (e.g. determining feasibility of new research)
- ☐ Pooled or meta-analyses
- ☐ Mathematical modeling
- ☐ Training artificial intelligence algorithms
- ☐ Reanalysis to verify research findings
- ☐ Secondary analysis (including generating/testing new hypotheses)
- ☐ Baseline data for new study
- ☐ Teaching
- ☐ Literature review
- ☐ Application for registration of a drug/medical device
- ☐ Health-related policy/guideline development
- ☐ Other

You selected Other, please specify:

____________________

**5) What outputs were generated from the data?** (*Select as many categories as appropriate*
*)*

- ☐ Publication
- ☐ Presentation (e.g. conference talk, poster, seminar, webinar)
- ☐ Thesis or Dissertation
- ☐ Report
- ☐ Book chapter
- ☐ Validation of mathematical model
- ☐ Training Artificial Intelligence algorithm
- ☐ Validation of software code

- [ ] Blog post
- [ ] Social media post
- [ ] Other
- [ ] No outputs were generated from use of this data

You selected Other, please specify:

Indicate total number of ***Publications*** based on the data.

- ( ) 1-4
- ( ) 5-10
- ( ) More than 10

Indicate total number of ***Presentations*** made from this data.

- ( ) 1-4
- ( ) 5-10
- ( ) More than 10

Indicate total number of ***Theses or Dissertations*** obtained from use of this data.

- ( ) 1-4
- ( ) 5-10
- ( ) More than 10

Indicate total number of ***Reports*** generated.

- ( ) 1-4
- ( ) 5-10

- More than 10

Indicate total number of ***Book chapters*** based on this data.

- 1-4
- 5-10
- More than 10

Indicate total number of ***mathematical models*** *validated.*

- 1-4
- 5-10
- More than 10

Indicate total number of ***artifical intelligence algorithms*** trained*.*

- 1-4
- 5-10
- More than 10

Indicate total number of ***software code snippets*** validated.

- 1-4
- 5-10
- More than 10

Indicate total number of ***Blog posts*** based on this data.

- 1-4
- 5-10
- More than 10

Indicate total number of ***Social media posts*** based on this data.

- 1-4
- 5-10
- More than 10

Indicate total number of ***outputs mentioned under 'Others'***.

- 1-4
- 5-10
- More than 10

**6) What were the outcomes of using the data ?** *(tick all the apply)*

- Validated or verified results from existing publication
- Correction or retraction of an existing publication
- MSc or PhD obtained
- New job created, e.g. internship, new analyst hired
- Career progression for me or my peers
- Increased citations and visibility for me/my institution
- Studies using secondary data as baseline were launched
- Design of a new study was influenced by results of the analysis
- New/increased collaboration with other researchers
- Invited to speak/contribute in expert forum
- Mention by authoritative body, e.g. Government body, WHO
- Findings included in health policy/treatment guidelines
- Registration of new drug or medical device
- Funding for my research or my institution
- Financial benefit from registration of drug or medical device

- ☐ Personal financial benefit, e.g. consultancy payment for secondary analysis
- ☐ Other
- ☐ None

You selected Other, please specify:

**7) Common issues experienced while *obtaining* shared datasets are listed below. In your experience, what impact did these issues have on your planned work?**

| | ☐ *Required* | | | | |
|---|---|---|---|---|---|
| | Did not encounter this issue | No impact | Low impact | Moderate impact | High impact |
| Difficulty finding relevant data | ☐ | ☐ | ☐ | ☐ | ☐ |
| Data not available at time of publication of research findings | ☐ | ☐ | ☐ | ☐ | ☐ |
| Unclear process for accessing the data | ☐ | ☐ | ☐ | ☐ | ☐ |
| The process or the required documentation is too laborious | ☐ | ☐ | ☐ | ☐ | ☐ |
| Data no longer exists on repository | ☐ | ☐ | ☐ | ☐ | ☐ |
| Slow or no response from data provider | ☐ | ☐ | ☐ | ☐ | ☐ |
| Ethical, legal or privacy restrictions with data | ☐ | ☐ | ☐ | ☐ | ☐ |
| Denied access to data | ☐ | ☐ | ☐ | ☐ | ☐ |
| Cost of data was prohibitive | ☐ | ☐ | ☐ | ☐ | ☐ |
| Data was provided with restrictions | ☐ | ☐ | ☐ | ☐ | ☐ |

Other problem not listed in the table above. (*please specify the problem and impact on your planned work)*

**8) Common difficulties experienced while *using* shared datasets are listed below. In your experience, what impact did these issues have on your planned work?**

| | *Required* | | | | |
|---|---|---|---|---|---|
| | Did not encounter this issue | No impact | Low impact | Moderate impact | High impact |
| Data variables needed were not collected in dataset | ☐ | ☐ | ☐ | ☐ | ☐ |
| Unusable data format or structure | ☐ | ☐ | ☐ | ☐ | ☐ |
| Errors or inconsistencies in data | ☐ | ☐ | ☐ | ☐ | ☐ |
| Incompleteness of data (many values were missing) | ☐ | ☐ | ☐ | ☐ | ☐ |
| Difficulty understanding the data | ☐ | ☐ | ☐ | ☐ | ☐ |
| Inappropriate study design | ☐ | ☐ | ☐ | ☐ | ☐ |
| Insufficient data (e.g. sample size too small) | ☐ | ☐ | ☐ | ☐ | ☐ |
| Data was in a different language | ☐ | ☐ | ☐ | ☐ | ☐ |
| Limited or no metadata (data dictionary, protocol, statistical analysis plan) | ☐ | ☐ | ☐ | ☐ | ☐ |
| Lack of resources to use the data (e.g. analysis, software, hardware, data curation) | ☐ | ☐ | ☐ | ☐ | ☐ |

Other problem not listed in the table above *(please specify the problem and impact on your planned work)*

**9) What kind of support or resources would enable you to access and use data**

**collected by other researchers more effectively?**

| | 1=least helpful, 5=most helpful | | | | |
|---|---|---|---|---|---|
| | 1 | 2 | 3 | 4 | 5 |
| Repositories : where to find relevant data | ☐ | ☐ | ☐ | ☐ | ☐ |
| Data licensing: terms and conditions for data use | ☐ | ☐ | ☐ | ☐ | ☐ |
| Analysis: appropriate methods and tools to assemble and use data | ☐ | ☐ | ☐ | ☐ | ☐ |
| Research etiquette: how to use data responsibly (authorship, attribution acknowledgement, intellectual property ) | ☐ | ☐ | ☐ | ☐ | ☐ |
| Legal assistance: negotiating and executing data sharing agreements | ☐ | ☐ | ☐ | ☐ | ☐ |
| Financial assistance: where data comes at a cost | ☐ | ☐ | ☐ | ☐ | ☐ |

Other*, please specify*

## ABOUT YOU

**10) What is your primary research discipline ?**

You selected Other, please specify:

**11) In which country is your employer located?** *(if for example, you are employed by a university in Sweden and work at a field site in Indonesia, please select Sweden as your employer's country)*

**12) What is the primary nature of your organisation?**

- University or Academic Research Organisation
- Government or public institution
- Non-Governmental or Faith-Based Organisation
- Commercial organization (e.g. pharmaceutical company)
- Ethical Review Committee
- Regulatory Authority
- Research Funder
- Other

You selected Other, please specify:

**13) What is your main job title/ role?**

More info

- Clinical researcher
- Statistician
- Epidemiologist
- Data Manager
- Data Scientist

- Bioinformatician
- Research support professional
- Other

You selected Other, please specify:

[ ]

**13a) Which of these best describe you?**

☐ More info

- Senior researcher
- Mid-career researcher
- Early career researcher
- Graduate student

**14) What is your age group ?**

[ ]

**15) What is your gender ?**

- Male
- Female
- Other
- Prefer not to say

16) Anything else you would like to tell us?

**1) What is the main reason for not using data collected by other researchers?**

- ○ I do not need to use other's data for my work
- ○ I could not find data relevant for my project
- ○ I could not access the data
- ○ I had difficulty using the data
- ○ Other

You selected Other, please specify:

[ ]

**1a) Common issues experienced while *obtaining* shared datasets are listed below. In your experience, what impact did these issues have on your project ?**

| | Did not encounter this issue | No impact | Low impact | Moderate impact | High impact |
|---|---|---|---|---|---|
| Difficulty finding relevant data | ☐ | ☐ | ☐ | ☐ | ☐ |
| Data not available at time of publication of research findings | ☐ | ☐ | ☐ | ☐ | ☐ |
| Unclear process for accessing the data | ☐ | ☐ | ☐ | ☐ | ☐ |
| The process or required documentation is too laborious | ☐ | ☐ | ☐ | ☐ | ☐ |
| Data no longer exists on repository | ☐ | ☐ | ☐ | ☐ | ☐ |

| | | | | | |
|---|---|---|---|---|---|
| Slow or no response from data provider | ☐ | ☐ | ☐ | ☐ | ☐ |
| Ethical, legal or privacy restrictions with data | ☐ | ☐ | ☐ | ☐ | ☐ |
| Denied access to data | ☐ | ☐ | ☐ | ☐ | ☐ |
| Cost of data was prohibitive | ☐ | ☐ | ☐ | ☐ | ☐ |
| Data was provided with restrictions | ☐ | ☐ | ☐ | ☐ | ☐ |

Other issue not listed in the table above *(please specify the problem and impact on your planned work)*

**1a) Common difficulties experienced while *using* shared datasets are listed below.In your experience,what impact did these issues have on your project?**

| | Did not encounter this issue | No impact | Low impact | Moderate impact | High impact |
|---|---|---|---|---|---|
| Data variables needed were not collected in dataset | ☐ | ☐ | ☐ | ☐ | ☐ |
| Unusable data format or structure | ☐ | ☐ | ☐ | ☐ | ☐ |
| Errors or inconsistencies in data | ☐ | ☐ | ☐ | ☐ | ☐ |
| Incompleteness of data (many values were missing) | ☐ | ☐ | ☐ | ☐ | ☐ |

| | | | | | |
|---|---|---|---|---|---|
| Difficulty understanding the data | ☐ | ☐ | ☐ | ☐ | ☐ |
| Inappropriate study design | ☐ | ☐ | ☐ | ☐ | ☐ |
| Insufficient data (e.g. sample size too small) | ☐ | ☐ | ☐ | ☐ | ☐ |
| Data was in a different language | ☐ | ☐ | ☐ | ☐ | ☐ |
| Limited or no metadata (data dictionary, protocol, statistical analysis plan) | ☐ | ☐ | ☐ | ☐ | ☐ |
| Lack of resources to use the data (e.g. analysis, software, hardware, data curation) | ☐ | ☐ | ☐ | ☐ | ☐ |

Other issue not listed in the table above *(please specify the problem and impact on your planned work)*

**2) What kind of support or resources would enable you to access and use data collected by other researchers more effectively?**

| | **1=least helpful, 5=most helpful** *Required* | | | | |
|---|---|---|---|---|---|
| | 1 | 2 | 3 | 4 | 5 |
| Repositories: where to find relevant data | ☐ | ☐ | ☐ | ☐ | ☐ |

| | | | | | |
|---|---|---|---|---|---|
| Data licensing: terms and conditions for data use | ☐ | ☐ | ☐ | ☐ | ☐ |
| Analysis: appropriate methods and tools to assemble and use the data | ☐ | ☐ | ☐ | ☐ | ☐ |
| Research etiquette: how to use data responsibly (authorship, attribution acknowledgement, intellectual property ) | ☐ | ☐ | ☐ | ☐ | ☐ |
| Legal assistance: negotiating and executing data sharing agreements | ☐ | ☐ | ☐ | ☐ | ☐ |
| Financial assistance: where data comes at a cost | ☐ | ☐ | ☐ | ☐ | ☐ |

Other, *please specify*

## ABOUT YOU

**3) What is your primary research discipline ?**

You selected Other, please specify:

**4) In which country is your employer located?** *(if for example, you are employed by a university in Sweden and work at a field site in Indonesia, please select Sweden as your employer's country)*

**5) What is the primary nature of your organisation?**

- University or Academic Research Organisation
- Government or public research institution
- Non-Governmental or Faith-Based Organisation
- Commercial organization (e.g. pharmaceutical company)
- Ethical Review Committee
- Regulatory Authority
- Research Funder
- Other

You selected Other, please specify:

**6) Which of these titles best describe you ?**

More info

- Non-academic professional
- Senior researcher
- Mid career researcher
- Early career researcher
- Graduate student
- Other

You selected Other, please specify:

### 7) What is your age group?

### 8) What is your gender ?

- Male
- Female
- Other
- Prefer not to say

### 9) Anything else you would like to tell us?

# Thank you for completing the survey

If you have any questions about this project, please email reuse@tropmedres.ac

---

## Key for selection options

**5 - 3) How many times have you requested data in the last 5 years ? (*if you cannot remember the exact number of requests,you may provide an estimate*)**

1
2
3
4
5
More than 5

**12 - 10) What is your primary research discipline ?**

Infectious Diseases
Global Health/Public Health
Clinical laboratory sciences
Clinical immunology
Clinical microbiology
Epidemiology
Molecular genetics
Parasitology
Dental science
Dermatology
Gynecology
Neurology
Nursing
Histology
Other

**13 - 11) In which country is your employer located? (*if for example, you are employed by a university in Sweden and work at a field site in Indonesia, please select Sweden as your employer's country)***

Afghanistan

Akrotiri
Albania
Algeria
American Samoa
Andorra
Angola
Anguilla
Antarctica
Antigua and Barbuda
Argentina
Armenia
Aruba
Ashmore and Cartier Islands
Australia
Austria
Azerbaijan
Bahamas, The
Bahrain
Bangladesh
Barbados
Bassas da India
Belarus
Belgium
Belize
Benin
Bermuda
Bhutan
Bolivia
Bosnia and Herzegovina
Botswana
Bouvet Island
Brazil
British Indian Ocean Territory
British Virgin Islands
Brunei
Bulgaria
Burkina Faso
Burma
Burundi
Cambodia

Cameroon
Canada
Cape Verde
Cayman Islands
Central African Republic
Chad
Chile
China
Christmas Island
Clipperton Island
Cocos (Keeling) Islands
Colombia
Comoros
Congo, Democratic Republic of the
Congo, Republic of the
Cook Islands
Coral Sea Islands
Costa Rica
Cote d'Ivoire
Croatia
Cuba
Cyprus
Czech Republic
Denmark
Dhekelia
Djibouti
Dominica
Dominican Republic
Ecuador
Egypt
El Salvador
Equatorial Guinea
Eritrea
Estonia
Ethiopia
Europa Island
Falkland Islands (Islas Malvinas)
Faroe Islands
Fiji
Finland

France
French Guiana
French Polynesia
French Southern and Antarctic Lands
Gabon
Gambia, The
Gaza Strip
Georgia
Germany
Ghana
Gibraltar
Glorioso Islands
Greece
Greenland
Grenada
Guadeloupe
Guam
Guatemala
Guernsey
Guinea
Guinea-Bissau
Guyana
Haiti
Heard Island and McDonald Islands
Holy See (Vatican City)
Honduras
Hong Kong
Hungary
Iceland
India
Indonesia
Iran
Iraq
Ireland
Isle of Man
Israel
Italy
Jamaica
Jan Mayen
Japan

Jersey
Jordan
Juan de Nova Island
Kazakhstan
Kenya
Kiribati
Korea, North
Korea, South
Kuwait
Kyrgyzstan
Laos
Latvia
Lebanon
Lesotho
Liberia
Libya
Liechtenstein
Lithuania
Luxembourg
Macau
Macedonia
Madagascar
Malawi
Malaysia
Maldives
Mali
Malta
Marshall Islands
Martinique
Mauritania
Mauritius
Mayotte
Mexico
Micronesia, Federated States of
Moldova
Monaco
Mongolia
Montenegro
Montserrat
Morocco

Mozambique
Namibia
Nauru
Navassa Island
Nepal
Netherlands
Netherlands Antilles
New Caledonia
New Zealand
Nicaragua
Niger
Nigeria
Niue
Norfolk Island
Northern Mariana Islands
Norway
Oman
Pakistan
Palau
Panama
Papua New Guinea
Paracel Islands
Paraguay
Peru
Philippines
Pitcairn Islands
Poland
Portugal
Puerto Rico
Qatar
Reunion
Romania
Russia
Rwanda
Saint Helena
Saint Kitts and Nevis
Saint Lucia
Saint Pierre and Miquelon
Saint Vincent and the Grenadines
Samoa

San Marino
Sao Tome and Principe
Saudi Arabia
Senegal
Serbia
Seychelles
Sierra Leone
Singapore
Slovakia
Slovenia
Solomon Islands
Somalia
South Africa
South Georgia and the South Sandwich Islands
Spain
Spratly Islands
Sri Lanka
Sudan
Suriname
Svalbard
Swaziland
Sweden
Switzerland
Syria
Taiwan
Tajikistan
Tanzania
Thailand
Timor-Leste
Togo
Tokelau
Tonga
Trinidad and Tobago
Tromelin Island
Tunisia
Turkey
Turkmenistan
Turks and Caicos Islands
Tuvalu
Uganda

Ukraine
United Arab Emirates
United Kingdom
United States
Uruguay
Uzbekistan
Vanuatu
Venezuela
Vietnam
Virgin Islands
Wake Island
Wallis and Futuna
West Bank
Western Sahara
Yemen
Zambia
Zimbabwe

**16 - 14) What is your age group ?**

18-24
25-34
35-44
45-54
55-64
65-74
75 or older
Prefer not to say

**21 - 3) What is your primary research discipline ?**

Infectious Diseases
Global Health/Public Health
Clinical laboratory sciences
Clinical immunology
Clinical microbiology
Epidemiology
Molecular genetics
Parasitology
Dental science
Dermatology

Gynecology
Neurology
Nursing
Histology
Other

**22 - 4) In which country is your employer located?** ***(if for example, you are employed by a university in Sweden and work at a field site in Indonesia, please select Sweden as your employer's country)***

Afghanistan
Akrotiri
Albania
Algeria
American Samoa
Andorra
Angola
Anguilla
Antarctica
Antigua and Barbuda
Argentina
Armenia
Aruba
Ashmore and Cartier Islands
Australia
Austria
Azerbaijan
Bahamas, The
Bahrain
Bangladesh
Barbados
Bassas da India
Belarus
Belgium
Belize
Benin
Bermuda
Bhutan
Bolivia
Bosnia and Herzegovina
Botswana

Bouvet Island
Brazil
British Indian Ocean Territory
British Virgin Islands
Brunei
Bulgaria
Burkina Faso
Burma
Burundi
Cambodia
Cameroon
Canada
Cape Verde
Cayman Islands
Central African Republic
Chad
Chile
China
Christmas Island
Clipperton Island
Cocos (Keeling) Islands
Colombia
Comoros
Congo, Democratic Republic of the
Congo, Republic of the
Cook Islands
Coral Sea Islands
Costa Rica
Cote d'Ivoire
Croatia
Cuba
Cyprus
Czech Republic
Denmark
Dhekelia
Djibouti
Dominica
Dominican Republic
Ecuador
Egypt

El Salvador
Equatorial Guinea
Eritrea
Estonia
Ethiopia
Europa Island
Falkland Islands (Islas Malvinas)
Faroe Islands
Fiji
Finland
France
French Guiana
French Polynesia
French Southern and Antarctic Lands
Gabon
Gambia, The
Gaza Strip
Georgia
Germany
Ghana
Gibraltar
Glorioso Islands
Greece
Greenland
Grenada
Guadeloupe
Guam
Guatemala
Guernsey
Guinea
Guinea-Bissau
Guyana
Haiti
Heard Island and McDonald Islands
Holy See (Vatican City)
Honduras
Hong Kong
Hungary
Iceland
India

Indonesia
Iran
Iraq
Ireland
Isle of Man
Israel
Italy
Jamaica
Jan Mayen
Japan
Jersey
Jordan
Juan de Nova Island
Kazakhstan
Kenya
Kiribati
Korea, North
Korea, South
Kuwait
Kyrgyzstan
Laos
Latvia
Lebanon
Lesotho
Liberia
Libya
Liechtenstein
Lithuania
Luxembourg
Macau
Macedonia
Madagascar
Malawi
Malaysia
Maldives
Mali
Malta
Marshall Islands
Martinique
Mauritania

Mauritius
Mayotte
Mexico
Micronesia, Federated States of
Moldova
Monaco
Mongolia
Montenegro
Montserrat
Morocco
Mozambique
Namibia
Nauru
Navassa Island
Nepal
Netherlands
Netherlands Antilles
New Caledonia
New Zealand
Nicaragua
Niger
Nigeria
Niue
Norfolk Island
Northern Mariana Islands
Norway
Oman
Pakistan
Palau
Panama
Papua New Guinea
Paracel Islands
Paraguay
Peru
Philippines
Pitcairn Islands
Poland
Portugal
Puerto Rico
Qatar

Reunion
Romania
Russia
Rwanda
Saint Helena
Saint Kitts and Nevis
Saint Lucia
Saint Pierre and Miquelon
Saint Vincent and the Grenadines
Samoa
San Marino
Sao Tome and Principe
Saudi Arabia
Senegal
Serbia
Seychelles
Sierra Leone
Singapore
Slovakia
Slovenia
Solomon Islands
Somalia
South Africa
South Georgia and the South Sandwich Islands
Spain
Spratly Islands
Sri Lanka
Sudan
Suriname
Svalbard
Swaziland
Sweden
Switzerland
Syria
Taiwan
Tajikistan
Tanzania
Thailand
Timor-Leste
Togo

Tokelau
Tonga
Trinidad and Tobago
Tromelin Island
Tunisia
Turkey
Turkmenistan
Turks and Caicos Islands
Tuvalu
Uganda
Ukraine
United Arab Emirates
United Kingdom
United States
Uruguay
Uzbekistan
Vanuatu
Venezuela
Vietnam
Virgin Islands
Wake Island
Wallis and Futuna
West Bank
Western Sahara
Yemen
Zambia
Zimbabwe

**25 - 7) What is your age group?**

18-24
25-34
35-44
45-54
55-64
65-74
75 or older
Prefer not to say
