# Supplementary material for: Investigating the Secondary Use of Clinical Research Data: Protocol for a Mixed Methods Study
Source: JMIR Res Protoc. 2023 Mar 6;12:e44875. doi: 10.2196/44875 (PMC10028503; doi:10.2196/44875)
Supplement: Multimedia Appendix 2 [file resprot_v12i1e44875_app2.pdf]

Esta encuesta forma parte de un estudio dirigido por investigadores de la Universidad de Oxford para comprender si los investigadores médicos utilizan los datos recopilados por otros y de qué manera lo hacen. Aunque no utilice datos recopilados por otros, sus comentarios son útiles para este estudio. Las respuestas son anónimas. Le llevará aproximadamente **3 -7** minutos completar la encuesta.

¿Cuál es el objetivo de esta encuesta?
Cada vez es más habitual compartir los datos obtenidos de estudios de investigación clínica. Queremos saber si se reutilizan los conjuntos de datos compartidos y de qué forma se utilizan, qué dificultades existen para acceder a los datos y reutilizarlos, y qué impacto ha tenido la reutilización de datos en la investigación científica y la salud pública en general.

¿Cuáles son los posibles beneficios de esta investigación?
Los datos de este estudio ayudarán a definir qué medidas deben priorizarse para aumentar la utilización efectiva de datos secundarios. Prevemos que una mayor reutilización de los datos redundará en una mayor calidad y transparencia de la ciencia, en una mejora de la salud pública y de los resultados de los pacientes, así como en una mayor rentabilidad de las inversiones en investigación.

Equipo de estudio e información
El estudio está dirigido por investigadores de la Universidad de la Universidad de Oxford y de la Unidad de Investigación de Medicina Tropical de Mahidol, con colaboradores en el Reino Unido, Kenia y Vietnam. El estudio ha sido aprobado por el Comité Ético de Investigación Tropical de Oxford (OxTREC, por sus siglas en inglés) número de referencia: 568-20.

Protección de datos
Al completar la encuesta, habrá proporcionado información sobre usted («datos personales»). La normativa de protección de datos exige que indiquemos el fundamento jurídico para el tratamiento de su información personal. En el caso de la investigación, se trata de «una tarea de interés público». La Universidad de Oxford es el controlador de datos y es responsable de cuidar su información y utilizarla de conformidad con el Reglamento General de Protección de Datos y la legislación de protección de datos asociada. Sus datos se conservarán de forma segura de conformidad con las políticas y procedimientos de la Universidad. Encontrará más información en el sitio web de la Universidad sobre Seguridad de la Información. Puede consultar toda la información sobre sus derechos en relación con sus datos personales aquí.

Los resultados de este proyecto de investigación se difundirán mediante informes de investigación, publicaciones/artículos y presentaciones. Tratamos sus datos para estos fines solo porque usted nos ha dado su consentimiento para hacerlo, marcando la casilla correspondiente. Sus respuestas serán anónimas, ya que la encuesta no recoge su nombre, dirección de correo electrónico o dirección IP. Por ello, no podremos retirar sus respuestas a posteriori, una vez que las haya enviado. Si abandona encuesta antes de enviar sus respuestas, sus datos no se almacenarán.

Contacto
Si desea plantear cualquier pregunta o duda sobre el uso que hacemos de sus datos, póngase en contacto con nosotros escribiendo a la dirección de correo electrónico *reuse@tropmedres.ac* o llamando al número de teléfono +66 02 203 6333 Ext 8302.
Si desea ponerse en contacto con alguien independiente del equipo del estudio, puede ponerse en contacto con el Comité de Ética de la Investigación Tropical de Oxford (OxTREC) escribiendo a la dirección de correo electrónico: *oxtrec@admin.ox.ac.uk.*

Protección de datos

Información adicional

Doy mi consentimiento para participar en la encuesta. Entiendo que mi participación es voluntaria y que soy libre de dejar de participar en cualquier momento.

¿Ha utilizado en alguna ocasión datos de investigación clínica compartidos por otros investigadores?

- Sí
- No

1) ¿Qué tipos de datos, compartidos por otros investigadores, ha utilizado?
(seleccione todas las respuestas que correspondan)

- ☐ Datos de ensayos clínicos
- ☐ Encuestas transversales de salud
- ☐ Datos de cohortes de observación
- ☐ Datos de vigilancia
- ☐ Datos administrativos
- ☐ Registros de pacientes/enfermedades
- ☐ Datos farmacológicos
- ☐ Datos de biología molecular
- ☐ Datos ómicos (genómica, transcriptómica, proteómica, epigenómica, metabolómica)
- ☐ Datos de ciencias sociales
- ☐ Datos de economía del sector sanitario
- ☐ Datos cualitativos
- ☐ Otros

Ha seleccionado datos cualitativos u «Otros». Por favor, especifique qué tipo de investigación generó los datos

2) ¿Cómo accedió a los conjuntos de datos? *(seleccione todas las respuestas que correspondan)*

- ☐ Mediante solicitud al recopilador/custodio de los datos
- ☐ Mediante descarga desde un sitio web/repositorio público
- ☐ Mediante solicitud a través de un Comité de Acceso a los Datos
- ☐ Otros

Ha seleccionado «Otros»; por favor, especifíquelo:

3) ¿Cuántas veces ha solicitado datos en los últimos 5 años? *(si no recuerda el número exacto de solicitudes, puede proporcionar una estimación)*

En 2017

En 2018

En 2019

En 2020

En 2021

4) ¿Para qué utilizó los datos? *(seleccione todas las respuestas que correspondan)*

- ☐ Para planificar y diseñar un nuevo estudio (por ejemplo, determinar la viabilidad de una nueva investigación)
- ☐ Agrupados o metaanálisis
- ☐ Modelización matemática
- ☐ Entrenamiento de algoritmos de inteligencia artificial
- ☐ Repetición de análisis para verificar los resultados de la investigación
- ☐ Análisis secundario (incluida la generación/prueba de nuevas hipótesis)
- ☐ Datos de referencia para un nuevo estudio
- ☐ Enseñanza
- ☐ Revisión bibliográfica
- ☐ Solicitud de registro de un medicamento/producto sanitario
- ☐ Desarrollo de políticas y directrices relacionadas con la salud
- ☐ Otros

Ha seleccionado «Otros»; por favor, especifíquelo:

5) ¿Qué resultados se generaron a partir de los datos? *(Seleccione tantas categorías como sea necesario)*

- ☐ Publicación
- ☐ Presentación (p. ej. charla en una conferencia, póster, seminario, seminario web)
- ☐ Tesis o disertación
- ☐ Informe
- ☐ Capítulo de un libro
- ☐ Validación de un modelo matemático
- ☐ Entrenamiento de un algoritmo de inteligencia artificial

- [ ] Validación de un código de software
- [ ] Publicación en un blog
- [ ] Publicación en redes sociales
- [ ] Otros
- [ ] No se generaron resultados a partir de la utilización de estos datos

Ha seleccionado «Otros»; por favor, especifíquelo:

Indique el número total de ***publicaciones*** basadas en los datos.

- 1-4
- 5-10
- Más de 10

Indique el número total de ***presentaciones*** elaboradas a partir de estos datos.

- 1-4
- 5-10
- Más de 10

Indique el número total de ***tesis o disertaciones*** obtenidas de la utilización de estos datos.

- 1-4
- 5-10
- Más de 10

Indique el número total de ***informes*** generados.

- 1-4
- 5-10

- Más de 10

Indique el número total de ***capítulos de libros*** escritos en base a estos datos.

- 1-4
- 5-10
- Más de 10

Indique el número total de ***modelos matemáticos*** *validados*.

- 1-4
- 5-10
- Más de 10

Indique el número total de ***algoritmos de inteligencia artificial*** entrenados.

- 1-4
- 5-10
- Más de 10

Indique el número total de ***fragmentos de código de software*** validados.

- 1-4
- 5-10
- Más de 10

Indique el número total de ***publicaciones en un blog*** escritas en base a estos datos.

- 1-4
- 5-10
- Más de 10

Indique el número total de ***publicaciones en redes sociales*** escritas con base en estos datos.

- 1-4
- 5-10
- Más de 10

Indique el número total de ***resultados mencionados bajo la categoría «Otros»***.

- 1-4
- 5-10
- Más de 10

6) ¿Cuáles fueron los resultados de la utilización de los datos? *(seleccione todas las respuestas que correspondan)*

- Validación o verificación de resultados a partir de una publicación existente
- Corrección o retractación de una publicación existente
- Obtención de un máster o doctorado.
- Creación de un nuevo puesto de trabajo, p. ej. prácticas, contratación de un nuevo analista
- Progresión profesional para mí o mis compañeros
- Aumento de las citas y de la visibilidad para mí/la institución en la que trabajo
- Puesta en marcha de estudios que utilizaban datos secundarios como referencia
- El diseño de un nuevo estudio se vio influenciado por los resultados del análisis
- Nueva colaboración o incremento de la colaboración con otros investigadores
- Invitación a hablar/contribuir en un foro de expertos
- Mención por parte de un organismo autorizado, p. ej. un organismo gubernamental o la OMS
- Inclusión de las conclusiones en directrices de política sanitaria/tratamiento
- Registro de un nuevo medicamento o producto sanitario

- [ ] Financiación para mi investigación o para la institución en la que trabajo
- [ ] Beneficio económico del registro de un medicamento o producto sanitario
- [ ] Beneficio económico personal, p. ej. pago de consultoría para un análisis secundario
- [ ] Otros
- [ ] Ninguno

Ha seleccionado «Otros»; por favor, especifíquelo:

7) A continuación se enumeran los problemas más comunes que se experimentan al *obtener* conjuntos de datos compartidos. Según su experiencia, ¿qué repercusión han tenido estos problemas en el trabajo previsto?

	✳ *Obligatorio*				
	No me he enfrentado a este problema	No han tenido ninguna repercusión	Han tenido poca repercusión	Han tenido bastante repercusión	Han tenido mucha repercusión
Dificultad para encontrar datos relevantes	☐	☐	☐	☐	☐
Datos no disponibles en el momento de la publicación de los resultados de la investigación	☐	☐	☐	☐	☐
Poca claridad del proceso para acceder a los datos	☐	☐	☐	☐	☐
El proceso o la documentación requerida son demasiado laboriosos	☐	☐	☐	☐	☐
Los datos ya no existen en el repositorio	☐	☐	☐	☐	☐
Respuesta lenta o nula del proveedor de datos	☐	☐	☐	☐	☐
Restricciones éticas, legales o de privacidad con los datos	☐	☐	☐	☐	☐
Acceso a los datos denegado	☐	☐	☐	☐	☐
El coste de los datos era prohibitivo	☐	☐	☐	☐	☐
Los datos se proporcionaron con restricciones	☐	☐	☐	☐	☐

Otro problema no mencionado en la tabla anterior. *(por favor, especifique el problema y su repercusión en el trabajo previsto)*

8) A continuación se enumeran las dificultades más comunes que se experimentan al *utilizar* conjuntos de datos compartidos. Según su experiencia, ¿qué repercusión han tenido estos problemas en el trabajo previsto?

	✳ *Obligatorio*				
	No me he enfrentado a problema	No han tenido ninguna repercusión	Han tenido poca repercusión	Han tenido bastante repercusión	Han tenido mucha repercusión
Las variables de datos necesarias no estaban incluidas en el conjunto de datos	☐	☐	☐	☐	☐
Formato o estructura de datos inutilizables	☐	☐	☐	☐	☐
Errores o incoherencias en los datos	☐	☐	☐	☐	☐
Datos incompletos (faltaban muchos valores)	☐	☐	☐	☐	☐
Dificultad para entender los datos	☐	☐	☐	☐	☐
Diseño inadecuado del estudio	☐	☐	☐	☐	☐
Datos insuficientes (p. ej. tamaño de la muestra demasiado pequeño)	☐	☐	☐	☐	☐
Los datos estaban en otro idioma	☐	☐	☐	☐	☐
Metadatos limitados o inexistentes (diccionario de datos, protocolo, plan de análisis estadístico)	☐	☐	☐	☐	☐
Falta de recursos para utilizar los datos (p. ej. análisis, software, hardware, custodia de datos)	☐	☐	☐	☐	☐

Otro problema no mencionado en la tabla anterior. *(por favor, especifique el problema y su repercusión en el trabajo previsto)*

9) ¿Qué tipo de apoyo o recursos le permitirían acceder a los datos recopilados por otros investigadores y utilizarlos con mayor eficacia?

	1=de menor utilidad, 5=de mayor utilidad				
	1	2	3	4	5
Repositorios: dónde encontrar datos relevantes	☐	☐	☐	☐	☐
Licencia de los datos: términos y condiciones de uso de los datos	☐	☐	☐	☐	☐
Análisis: métodos y herramientas adecuados para reunir y utilizar los datos	☐	☐	☐	☐	☐
Protocolo de la investigación: cómo utilizar los datos de forma responsable (autoría, reconocimiento de la autoría, propiedad intelectual)	☐	☐	☐	☐	☐
Asistencia jurídica: negociación y ejecución de acuerdos de intercambio de datos	☐	☐	☐	☐	☐
Asistencia financiera: cuando los datos implican un coste	☐	☐	☐	☐	☐

Otros; por favor, *especifique cuáles:*

ACERCA DE USTED

10) ¿Cuál es su principal disciplina de investigación?

Ha seleccionado «Otros»; por favor, especifíquelo:

11) ¿En qué país se encuentra su empleador? *(si, por ejemplo, trabaja para una universidad en Suecia y desempeña su labor en un emplazamiento en Indonesia, seleccione Suecia como país de su empleador)*

12) ¿Cuál es la naturaleza principal de su organización?

- Universidad u organización de investigación académica
- Gobierno o institución pública
- Organización no gubernamental o religiosa
- Organización comercial (p. ej. una empresa farmacéutica)
- Comité de Revisión Ética
- Autoridad reguladora
- Financiador de la investigación
- Otros

Ha seleccionado «Otros»; por favor, especifíquelo:

13) ¿Cuál es su puesto o función principal?

Información adicional

- Investigador clínico
- Estadista
- Epidemiólogo
- Gestor de datos
- Científico de datos

- Bioinformático
- Profesional de apoyo a la investigación
- Otros

Ha seleccionado «Otros»; por favor, especifíquelo:

13a) ¿Cuál de las siguientes opciones le describe mejor?

Información adicional

- Investigador senior
- Investigador a mitad de carrera
- Investigador novel
- Estudiante de posgrado

14) ¿A qué grupo de edad pertenece?

15) ¿Es usted hombre o mujer?

- Hombre
- Mujer
- Otro
- Prefiero no responder

16) ¿Hay algo más que le gustaría compartir con nosotros?

1) ¿Cuál es el principal motivo por el que no ha utilizado datos recopilados por otros investigadores?

- No necesito utilizar datos de otros para mi trabajo
- No pude encontrar datos relevantes para mi proyecto
- No pude acceder a los datos
- Tuve dificultades a la hora de utilizar los datos
- Otros

Ha seleccionado «Otros»; por favor, especifíquelo:

1a) A continuación se enumeran los problemas más comunes que se experimentan al *obtener* conjuntos de datos compartidos. Según su experiencia, ¿qué repercusión han tenido estos problemas en su proyecto?

	No me he enfrentado a este problema	No han tenido ninguna repercusión	Han tenido poca repercusión	Han tenido bastante repercusión	Han tenido mucha repercusión
Dificultad para encontrar datos relevantes					
Datos no disponibles en el momento de la publicación de los resultados de la investigación					
Poca claridad del proceso para acceder a los datos					
El proceso o la documentación requerida son demasiado laboriosos					

Los datos ya no existen en el repositorio	☐	☐	☐	☐	☐
Respuesta lenta o nula del proveedor de datos	☐	☐	☐	☐	☐
Restricciones éticas, legales o de privacidad con los datos	☐	☐	☐	☐	☐
Acceso a los datos denegado	☐	☐	☐	☐	☐
El coste de los datos era prohibitivo	☐	☐	☐	☐	☐
Los datos se proporcionaron con restricciones	☐	☐	☐	☐	☐

Otro problema no incluido en la tabla anterior *(por favor, especifique el problema y su repercusión en el trabajo previsto)*

1a) A continuación se enumeran las dificultades más comunes que se experimentan al utilizar conjuntos de datos compartidos. Según su experiencia, ¿qué repercusión han tenido estos problemas en su proyecto?

	No me he enfrentado a este problema	No han tenido ninguna repercusión	Han tenido poca repercusión	Han tenido bastante repercusión	Han tenido mucha repercusión
Las variables de datos necesarias no estaban incluidas en el conjunto de datos	☐	☐	☐	☐	☐
Formato o estructura de datos inutilizables	☐	☐	☐	☐	☐

Errores o incoherencias en los datos	☐	☐	☐	☐	☐
Datos incompletos (faltaban muchos valores)	☐	☐	☐	☐	☐
Dificultad para entender los datos	☐	☐	☐	☐	☐
Diseño inadecuado del estudio	☐	☐	☐	☐	☐
Datos insuficientes (p. ej. tamaño de la muestra demasiado pequeño)	☐	☐	☐	☐	☐
Los datos estaban en otro idioma	☐	☐	☐	☐	☐
Metadatos limitados o inexistentes (diccionario de datos, protocolo, plan de análisis estadístico)	☐	☐	☐	☐	☐
Falta de recursos para utilizar los datos (p. ej. análisis, software, hardware, custodia de datos)	☐	☐	☐	☐	☐

Otro problema no incluido en la tabla anterior *(por favor, especifique el problema y su repercusión en el trabajo previsto)*

2) ¿Qué tipo de apoyo o recursos le permitirían acceder a los datos recopilados por otros investigadores y utilizarlos con mayor eficacia?

	**1=de menor utilidad, 5=de mayor utilidad** ✳ *Obligatorio*				
	1	2	3	4	5
Repositorios: dónde encontrar datos relevantes	☐	☐	☐	☐	☐
Licencias de datos: términos y condiciones de uso de los datos	☐	☐	☐	☐	☐
Análisis: métodos y herramientas adecuados para reunir y utilizar los datos	☐	☐	☐	☐	☐
Protocolo de la investigación: cómo utilizar los datos de forma responsable (autoría, reconocimiento de la autoría, propiedad intelectual)	☐	☐	☐	☐	☐
Asistencia jurídica: negociación y ejecución de acuerdos de intercambio de datos	☐	☐	☐	☐	☐
Asistencia financiera: cuando los datos implican un coste	☐	☐	☐	☐	☐

Otros; *por favor*, *especifique cuáles:*

ACERCA DE USTED

3) ¿Cuál es su principal disciplina de investigación?

Ha seleccionado «Otros»; por favor, especifíquelo:

4) ¿En qué país se encuentra su empleador? *(si, por ejemplo, trabaja para una universidad en Suecia y desempeña su labor en un emplazamiento en Indonesia, seleccione Suecia como país de su empleador)*

5) ¿Cuál es la naturaleza principal de su organización?

- Universidad u organización de investigación académica
- Gobierno o institución de investigación pública
- Organización no gubernamental o religiosa
- Organización comercial (p. ej. una empresa farmacéutica)
- Comité de Revisión Ética
- Autoridad reguladora
- Financiador de la investigación
- Otros

Ha seleccionado «Otros»; por favor, especifíquelo:

6) ¿Cuál de estos puestos le define mejor?

Información adicional

- Profesional no académico
- Investigador senior
- Investigador a mitad de carrera

- Investigador novel
- Estudiante de posgrado
- Otros

Ha seleccionado «Otros»; por favor, especifíquelo:

7) ¿A qué grupo de edad pertenece?

8) ¿Es usted hombre o mujer?

- Hombre
- Mujer
- Otro
- Prefiero no responder

9) ¿Hay algo más que le gustaría compartir con nosotros?

Gracias por participar en esta encuesta.

Si tiene alguna pregunta sobre este proyecto, envíe un correo electrónico a reuse@tropmedres.ac

---

Clave para las opciones de selección

5- 3) ¿Cuántas veces ha solicitado datos en los últimos 5 años? (*si no recuerda el número exacto de solicitudes, puede proporcionar una estimación*)

- 1
- 2
- 3
- 4
- 5
- Más de 5

12- 10) ¿Cuál es su principal disciplina de investigación?

- Enfermedades infecciosas
- Salud mundial/salud pública
- Ciencias de laboratorio clínico
- Inmunología clínica
- Microbiología clínica
- Epidemiología
- Genética molecular
- Parasitología
- Odontología
- Dermatología
- Ginecología
- Neurología
- Enfermería
- Histología
- Otros

13- 11) ¿En qué país se encuentra su empleador? (*si, por ejemplo, trabaja para una universidad en Suecia y desempeña su labor en un emplazamiento en Indonesia, seleccione Suecia como país de su empleador)*

- Afganistán

Acrotiri
Albania
Argelia
Samoa Americana
Andorra
Angola
Anguila
Antártida
Antigua y Barbuda
Argentina
Armenia
Aruba
Islas Ashmore y Cartier
Australia
Austria
Azerbaiyán
Bahamas
Baréin
Bangladesh
Barbados
Bassas da India
Bielorrusia
Bélgica
Belice
Benín
Las Bermudas
Bután
Bolivia
Bosnia y Herzegovina
Botsuana
Isla Bouvet
Brasil
Territorio Británico del Océano Índico
Islas Vírgenes Británicas
Brunéi
Bulgaria
Burkina Faso
Birmania
Burundi
Camboya

Camerún
Canadá
Cabo Verde
Islas Caimán
República Centroafricana
Chad
Chile
China
Isla Christmas
Isla de Clipperton
Islas Cocos
Colombia
Comoras
República Democrática del Congo
República del Congo
Islas Cook
Islas del Mar del Coral
Costa Rica
Costa de Marfil
Croacia
Cuba
Chipre
República Checa
Dinamarca
Dhekelia
Yibuti
Dominica
República Dominicana
Ecuador
Egipto
El Salvador
Guinea Ecuatorial
Eritrea
Estonia
Etiopía
Isla de Europa
Islas Malvinas
Islas Feroe
Fiyi
Finlandia

Francia
Guayana Francesa
Polinesia Francesa
Tierras Australes y Antárticas Francesas
Gabón
Gambia
Franja de Gaza
Georgia
Alemania
Ghana
Gibraltar
Islas Gloriosas
Grecia
Groenlandia
Granada
Guadalupe
Guam
Guatemala
Guernsey
Guinea
Guinea-Bissau
Guyana
Haití
Isla Heard e Islas McDonald
Santa Sede (Ciudad del Vaticano)
Honduras
Hong Kong
Hungría
Islandia
La India
Indonesia
Irán
Irak
Irlanda
Isla de Man
Israel
Italia
Jamaica
Jan Mayen
Japón

Jersey
Jordania
Isla Juan de Nova
Kazajstán
Kenia
Kiribati
Corea del Norte
Corea del Sur
Kuwait
Kirguistán
Laos
Letonia
Líbano
Lesoto
Liberia
Libia
Liechtenstein
Lituania
Luxemburgo
Macao
Macedonia
Madagascar
Malawi
Malasia
Maldivas
Mali
Malta
Islas Marshall
Martinica
Mauritania
Mauricio
Mayotte
México
Micronesia, Estados Federados de
Moldavia
Mónaco
Mongolia
Montenegro
Montserrat
Marruecos

Mozambique
Namibia
Nauru
Isla de Navaza
Nepal
Países Bajos
Antillas Neerlandesas
Nueva Caledonia
Nueva Zelanda
Nicaragua
Níger
Nigeria
Niue
Isla Norfolk
Islas Marianas del Norte
Noruega
Omán
Pakistán
Palaos
Panamá
Papúa Nueva Guinea
Islas Paracelso
Paraguay
Perú
Filipinas
Islas Pitcairn
Polonia
Portugal
Puerto Rico
Catar
Reunión
Rumanía
Rusia
Ruanda
Santa Helena
San Cristóbal y Nieves
Santa Lucía
San Pedro y Miquelón
San Vicente y las Granadinas
Samoa

San Marino
Santo Tomé y Príncipe
Arabia Saudí
Senegal
Serbia
Seychelles
Sierra Leona
Singapur
Eslovaquia
Eslovenia
Islas Salomón
Somalia
Sudáfrica
Georgia del Sur e Islas Sandwich del Sur
España
Islas Spratly
Sri Lanka
Sudán
Surinam
Svalbard
Suazilandia
Suecia
Suiza
Siria
Taiwán
Tayikistán
Tanzania
Tailandia
Timor Oriental
Togo
Tokelau
Tonga
Trinidad y Tobago
Isla de Tromelin
Túnez
Turquía
Turkmenistán
Islas Turcas y Caicos
Tuvalu
Uganda

- Ucrania
- Emiratos Árabes Unidos
- Reino Unido
- Estados Unidos
- Uruguay
- Uzbekistán
- Vanuatu
- Venezuela
- Vietnam
- Islas Vírgenes
- Isla de Wake
- Wallis y Futuna
- Cisjordania
- Sáhara Occidental
- Yemen
- Zambia
- Zimbabue

16- 14) ¿A qué grupo de edad pertenece?

- 18-24
- 25-34
- 35-44
- 45-54
- 55-64
- 65-74
- 75 años o más
- Prefiero no responder

21- 3) ¿Cuál es su principal disciplina de investigación?

- Enfermedades infecciosas
- Salud mundial/salud pública
- Ciencias de laboratorio clínico
- Inmunología clínica
- Microbiología clínica
- Epidemiología
- Genética molecular
- Parasitología
- Odontología
- Dermatología

Ginecología
Neurología
Enfermería
Histología
Otros

22- 4) ¿En qué país se encuentra su empleador? ***(si, por ejemplo, trabaja para una universidad en Suecia y desempeña su labor en un emplazamiento en Indonesia, seleccione Suecia como país de su empleador)***

Afganistán
Acrotiri
Albania
Argelia
Samoa Americana
Andorra
Angola
Anguila
Antártida
Antigua y Barbuda
Argentina
Armenia
Aruba
Islas Ashmore y Cartier
Australia
Austria
Azerbaiyán
Bahamas
Bahréin
Bangladesh
Barbados
Bassas da India
Bielorrusia
Bélgica
Belice
Benín
Las Bermudas
Bután
Bolivia
Bosnia y Herzegovina
Botsuana

Isla Bouvet
Brasil
Territorio Británico del Océano Índico
Islas Vírgenes Británicas
Brunéi
Bulgaria
Burkina Faso
Birmania
Burundi
Camboya
Camerún
Canadá
Cabo Verde
Islas Caimán
República Centroafricana
Chad
Chile
China
Isla Christmas
Isla de Clipperton
Islas Cocos
Colombia
Comoras
República Democrática del Congo
República del Congo
Islas Cook
Islas del Mar del Coral
Costa Rica
Costa de Marfil
Croacia
Cuba
Chipre
República Checa
Dinamarca
Dhekelia
Yibuti
Dominica
República Dominicana
Ecuador
Egipto

El Salvador
Guinea Ecuatorial
Eritrea
Estonia
Etiopía
Isla de Europa
Islas Malvinas
Islas Feroe
Fiyi
Finlandia
Francia
Guayana Francesa
Polinesia Francesa
Tierras Australes y Antárticas Francesas
Gabón
Gambia
Franja de Gaza
Georgia
Alemania
Ghana
Gibraltar
Islas Gloriosas
Grecia
Groenlandia
Granada
Guadalupe
Guam
Guatemala
Guernsey
Guinea
Guinea-Bissau
Guyana
Haití
Isla Heard e Islas McDonald
Santa Sede (Ciudad del Vaticano)
Honduras
Hong Kong
Hungría
Islandia
India

Indonesia
Irán
Irak
Irlanda
Isla de Man
Israel
Italia
Jamaica
Jan Mayen
Japón
Jersey
Jordania
Isla Juan de Nova
Kazajstán
Kenia
Kiribati
Corea del Norte
Corea del Sur
Kuwait
Kirguistán
Laos
Letonia
Líbano
Lesoto
Liberia
Libia
Liechtenstein
Lituania
Luxemburgo
Macao
Macedonia
Madagascar
Malawi
Malasia
Maldivas
Mali
Malta
Islas Marshall
Martinica
Mauritania

Mauricio
Mayotte
México
Micronesia, Estados Federados de
Moldavia
Mónaco
Mongolia
Montenegro
Montserrat
Marruecos
Mozambique
Namibia
Nauru
Isla de Navaza
Nepal
Países Bajos
Antillas Neerlandesas
Nueva Caledonia
Nueva Zelanda
Nicaragua
Níger
Nigeria
Niue
Isla Norfolk
Islas Marianas del Norte
Noruega
Omán
Pakistán
Palaos
Panamá
Papúa Nueva Guinea
Islas Paracelso
Paraguay
Perú
Filipinas
Islas Pitcairn
Polonia
Portugal
Puerto Rico
Catar

Reunión
Rumanía
Rusia
Ruanda
Santa Helena
San Cristóbal y Nieves
Santa Lucía
San Pedro y Miquelón
San Vicente y las Granadinas
Samoa
San Marino
Santo Tomé y Príncipe
Arabia Saudí
Senegal
Serbia
Seychelles
Sierra Leona
Singapur
Eslovaquia
Eslovenia
Islas Salomón
Somalia
Sudáfrica
Georgia del Sur e Islas Sandwich del Sur
España
Islas Spratly
Sri Lanka
Sudán
Surinam
Svalbard
Suazilandia
Suecia
Suiza
Siria
Taiwán
Tayikistán
Tanzania
Tailandia
Timor Oriental
Togo

Tokelau
Tonga
Trinidad y Tobago
Isla de Tromelin
Túnez
Turquía
Turkmenistán
Islas Turcas y Caicos
Tuvalu
Uganda
Ucrania
Emiratos Árabes Unidos
Reino Unido
Estados Unidos
Uruguay
Uzbekistán
Vanuatu
Venezuela
Vietnam
Islas Vírgenes
Isla de Wake
Wallis y Futuna
Cisjordania
Sáhara Occidental
Yemen
Zambia
Zimbabue

25- 7) ¿A qué grupo de edad pertenece?

18-24
25-34
35-44
45-54
55-64
65-74
75 años o más
Prefiero no responder
