# Supplementary material for: Investigating the Secondary Use of Clinical Research Data: Protocol for a Mixed Methods Study
Source: JMIR Res Protoc. 2023 Mar 6;12:e44875. doi: 10.2196/44875 (PMC10028503; doi:10.2196/44875)
Supplement: Multimedia Appendix 3 [file resprot_v12i1e44875_app3.pdf]

Cette enquête fait partie d'une étude menée par les chercheurs de l'Université d'Oxford en vue de comprendre si les chercheurs en médecine utilisent des données collectées par d'autres et de quelle manière. Même si vous n'utilisez pas de données collectées par d'autres personnes, vos informations sont utiles pour cette étude. Vos réponses sont anonymes. Cette enquête vous prendra **3 à 7** minutes.

Quel est l'objectif de la recherche ?
Le partage de données provenant d'études de recherche clinique devient de plus en plus la règle. Nous cherchons à comprendre si les ensembles de données partagés sont réutilisés et de quelle manière, quels sont les défis liés à l'accès et à la réutilisation des données, et quel impact la réutilisation des données a eu sur la recherche scientifique et la santé publique en général.

Quels sont les possibles avantages de cette recherche ?
Les données provenant de cette étude aideront à définir quelles mesures doivent être mises en œuvre pour rendre plus efficace l'utilisation des données secondaires. Nous prévoyons qu'une réutilisation accrue des données entraînera une amélioration de la qualité et de la transparence de la science, une amélioration de la santé publique et des résultats pour les patients ainsi qu'un meilleur retour sur investissement dans la recherche.

Équipe de l'étude et informations
L'étude est menée par des chercheurs de l'Université d'Oxford et de l'Unité de recherche en médecine tropicale Mahidol Oxford avec des collaborateurs au Royaume-Uni, au Kenya et au Vietnam. L'étude a été approuvée par le Comité d'éthique de la recherche tropicale de l'Université d'Oxford (OxTREC) ,numéro de référence : 568-20.

Protection des données
Au cours de la réalisation de l'enquête, vous aurez fourni des informations sur vous (les « données à caractère personnel »). La réglementation sur la protection des données exige que nous indiquions la base légale pour le traitement des informations vous concernant. Dans le cas d'une recherche, il s'agit d'une « mission d'intérêt général ». L'Université d'Oxford est le responsable du traitement et elle est tenue de surveiller vos informations et de les utiliser conformément au Règlement général sur la protection des données et à la législation connexe sur la protection des données. Vos données seront conservées en toute sécurité conformément aux politiques et aux procédures de l'université. D'autres information sont disponibles sur le site Web Sécurité des informations de l'université. Les informations sur vos droits en relation avec vos données personnelles sont expliquées ici.

Les conclusions de ce projet de recherche seront diffusées dans des rapports de recherche, des publications / articles et des présentations. Nous traitons vos données à ces fins uniquement, parce que vous nous avez donné votre consentement pour le faire en cochant la case appropriée. Vos réponses seront anonymes, car l'enquête ne collecte pas votre nom, votre adresse e-mail ou votre adresse IP. À cause de cela, nous ne serons pas en mesure de retirer vos réponses de manière rétrospective, après que vous les ayez soumises. Si vous quittez l'enquête avant de soumettre vos réponses, vos données ne seront pas conservées.

Contact
Si vous souhaitez poser des questions ou nous faire part de préoccupations sur notre utilisation de vos données, contactez-nous à l'adresse *reuse@tropmedres.ac* ou téléphonez-nous au numéro +66 02 203 6333 Poste 8302.
Si vous souhaitez contacter une personne ne faisant pas partie de l'équipe de l'étude, vous pouvez envoyer un courriel au Comité d'éthique de la recherche tropicale de l'Université d'Oxford (OxTREC) à cette adresse : oxtrec@admin.ox.ac.uk.

Protection des données

Plus d’informations

J’accepte de participer à l’enquête. Je comprends que ma participation est volontaire et que je suis libre de me retirer à tout moment

Avez-vous utilisé des données de recherche clinique qui ont été partagées par d’autres chercheurs ?

- Oui
- Non

1) Quels types de données, partagées par d’autres chercheurs, ont été utilisés ? *(Cochez toutes les réponses appropriées)*

- ☐ Données d’essais cliniques
- ☐ Enquêtes transversales sur la santé
- ☐ Données des cohortes observationnelles
- ☐ Données de surveillance
- ☐ Données administratives
- ☐ Registres de patients / maladies
- ☐ Données pharmacologiques
- ☐ Données de biologie moléculaire
- ☐ Données omiques (génomique, transcriptomique, protéomique, épigénomique, métabolomique)
- ☐ Données en sciences sociales
- ☐ Données en économie de la santé
- ☐ Données qualitatives
- ☐ Autre

Vous avez sélectionné des données Qualitatives ou « Autres ». Veuillez préciser quel genre de recherche a généré les données.

2) Comment avez-vous eu accès aux ensembles de données ? *(Cochez toutes les réponses appropriées)*

- ☐ Demande faite au collecteur / dépositaire des données
- ☐ Téléchargement depuis un site Web / service d’archivage des données
- ☐ Demande faite par le biais d’un Comité d’accès aux données
- ☐ Autre

Vous avez sélectionné « Autre », veuillez préciser :

3) Combien de fois avez-vous demandé des données au cours des 5 dernières années ? *(Si vous ne vous souvenez pas du nombre exact de demandes, vous pouvez fournir une estimation)*

En 2017

En 2018

En 2019

En 2020

En 2021

4) Pourquoi avez-vous utilisé les données ? *(Cochez toutes les réponses appropriées)*

- ☐ Planification et conception d'une nouvelle étude (par ex. déterminer la faisabilité d'une nouvelle recherche)
- ☐ Méta-analyses ou analyses regroupées
- ☐ Modélisation mathématique
- ☐ Algorithme d'entraînement de l'intelligence artificielle
- ☐ Réanalyse pour vérifier les résultats de la recherche
- ☐ Analyse secondaire (y compris générer / tester de nouvelles hypothèses)
- ☐ Données de base pour une nouvelle étude
- ☐ Enseignement
- ☐ Analyse documentaire
- ☐ Demande d'inscription d'un médicament / dispositif médical
- ☐ Élaboration de politiques / directives liées à la santé
- ☐ Autre

Vous avez sélectionné « Autre », veuillez préciser :

5) Quels résultats ont généré les données ? *(Sélectionnez autant de catégories que nécessaire)*

- ☐ Publication
- ☐ Présentation (par ex. conférence, affiche, séminaire, webinaire)
- ☐ Thèse ou dissertation
- ☐ Rapport
- ☐ Chapitre de livre
- ☐ Validation d'un modèle mathématique
- ☐ Algorithme d'entraînement de l'intelligence artificielle
- ☐ Validation d'un code logiciel

- [ ] Article de blog
- [ ] Publication sur les réseaux sociaux
- [ ] Autre
- [ ] Aucun résultat n’a été généré suite à l’utilisation de ces données

Vous avez sélectionné « Autre », veuillez préciser :

Indiquez le nombre total de ***Publications*** basées sur les données.

- 1-4
- 5-10
- Plus de 10

Indiquez le nombre total de ***Présentations*** effectuées à partir de ces données.

- 1-4
- 5-10
- Plus de 10

Indiquez le nombre total de ***Thèses ou de Dissertations*** provenant de l’utilisation de ces données.

- 1-4
- 5-10
- Plus de 10

Indiquez le nombre total de ***Rapports*** générés.

- 1-4
- 5-10

- Plus de 10

Indiquez le nombre total de ***Chapitres de livre*** basés sur les données.

- 1-4
- 5-10
- Plus de 10

Indiquez le nombre total de ***Modèles mathématiques*** *validés*.

- 1-4
- 5-10
- Plus de 10

Indiquez le nombre total d'***Algorithmes d'intelligence artificielle*** entraînés.

- 1-4
- 5-10
- Plus de 10

Indiquez le nombre total de ***Fragments de code logiciel*** validés.

- 1-4
- 5-10
- Plus de 10

Indiquez le nombre total d'***Articles de blog*** basés sur les données.

- 1-4
- 5-10
- Plus de 10

Indiquez le nombre total de ***Publications sur les réseaux sociaux*** basées sur les données.

- 1-4
- 5-10
- Plus de 10

Indiquez le nombre total de ***Résultats mentionnés sous « Autres ».***

- 1-4
- 5-10
- Plus de 10

6) Qu'est-ce que l'utilisation des données a eu comme résultat ? *(Cochez toutes les réponses appropriées)*

- Validation ou vérification de résultats provenant d'une publication existante
- Correction ou retrait d'une publication existante
- Obtention d'un doctorat ou d'un master en sciences
- Création d'un nouvel emploi, par ex. stage, embauche d'un nouvel analyste
- Progression de carrière pour moi ou mes pairs
- Plus de citations et de visibilité pour moi / mon institution
- Lancement d'études utilisant des données secondaires comme base
- Conception d'une nouvelle étude influencée par les résultats de l'analyse
- Collaboration nouvelle / accrue avec d'autres chercheurs
- Invitation à parler / contribuer à un forum d'experts
- Mention par un organisme faisant autorité, par ex. organisme gouvernemental, OMS
- Résultats inclus dans une politique de santé / les lignes directrices d'un traitement
- Enregistrement d'un nouveau médicament ou d'un dispositif médical
- Financement pour ma recherche ou mon institution

- [ ] Avantage financier tiré de l’enregistrement d’un médicament ou d’un dispositif médical
- [ ] Avantage financier personnel, par ex. paiement de services consultatifs pour une analyse secondaire
- [ ] Autre
- [ ] Aucun

Vous avez sélectionné « Autre », veuillez préciser :

7) Des problèmes courants rencontrés lors de *l'obtention* d'ensembles de données partagés sont énumérés ci-dessous. D'après ce que vous avez pu constater, quel impact ces problèmes ont-ils eu sur le travail que vous aviez prévu ?

	✳ *Exigé*				
	Je n'ai pas rencontré ce problème	Aucun impact	Faible impact	Impact modéré	Impact élevé
Difficulté à trouver des données pertinentes	☐	☐	☐	☐	☐
Données non disponibles au moment de la publication des résultats de la recherche	☐	☐	☐	☐	☐
Processus d'accès aux données manquant de clarté	☐	☐	☐	☐	☐
Processus ou documentation exigé(e) trop laborieux(se)	☐	☐	☐	☐	☐
Données n'existant plus dans le service d'archivage des données	☐	☐	☐	☐	☐
Réponse lente ou inexistante du fournisseur de données	☐	☐	☐	☐	☐
Restrictions d'ordre éthique, légal ou privé relatives aux données	☐	☐	☐	☐	☐
Accès aux données refusé	☐	☐	☐	☐	☐
Coût des données prohibitif	☐	☐	☐	☐	☐
Données fournies avec des restrictions	☐	☐	☐	☐	☐

Autre problème non répertorié dans le tableau ci-dessus. *(Veuillez préciser le problème et l'impact sur le travail que vous aviez prévu)*

8) Des problèmes courants rencontrés lors de *l'utilisation* d'ensembles de données partagés sont énumérés ci-dessous. D'après ce que vous avez pu constater, quel impact ces problèmes ont-ils eu sur le travail que vous aviez prévu ?

	✳ Exigé				
	Je n'ai pas rencontré ce problème	Aucun impact	Faible impact	Impact modéré	Impact élevé
Les variables de données nécessaires n'étaient pas collectées dans l'ensemble de données	☐	☐	☐	☐	☐
Format ou structure des données inutilisable	☐	☐	☐	☐	☐
Erreurs ou incohérences dans les données	☐	☐	☐	☐	☐
Données non complètes (beaucoup de valeurs manquantes)	☐	☐	☐	☐	☐
Difficulté à comprendre les données	☐	☐	☐	☐	☐
Conception d'étude inappropriée	☐	☐	☐	☐	☐
Données insuffisantes (par ex. trop petite taille de l'échantillon)	☐	☐	☐	☐	☐
Données fournies dans une autre langue	☐	☐	☐	☐	☐
Pas de méta données ou des données limitées (dictionnaire de données, protocole, plan d'analyse statistique)	☐	☐	☐	☐	☐
Manque de ressources pour utiliser les données (par ex. analyses, logiciel, matériel, conservation des données)	☐	☐	☐	☐	☐

Autre problème non répertorié dans le tableau ci-dessus. *(Veuillez préciser le problème et l'impact sur le travail que vous aviez prévu)*

9) Quel genre de support ou de ressources vous permettrait d'avoir accès et d'utiliser plus efficacement les données collectées par d'autres chercheurs ?

	1 = le moins utile, 5 = le plus utile				
	1	2	3	4	5
Services d'archivage des données : endroit où trouver les données pertinentes	☐	☐	☐	☐	☐
Octroi de licence de données : conditions générales de l'utilisation des données	☐	☐	☐	☐	☐
Analyse : méthodes et outils appropriés pour rassembler et utiliser les données	☐	☐	☐	☐	☐
Règles en matière de recherche : comment utiliser les données de manière responsable (paternité de l'œuvre, reconnaissance d'attribution, propriété intellectuelle)	☐	☐	☐	☐	☐
Assistance légale : négociation et conclusion des accords sur le partage des données	☐	☐	☐	☐	☐
Assistance financière : lorsque les données ont un coût	☐	☐	☐	☐	☐

Autre, *veuillez préciser*

QU'EN EST-IL DE VOUS ?

10) Quelle est votre principale discipline de recherche ?

Vous avez sélectionné « Autre », veuillez préciser :

11) Dans quel pays votre employeur est-il situé ? *(Si, par exemple, vous êtes employé(e) par une université située en Suède et que vous travaillez sur un site en Indonésie, sélectionnez Suède en tant que pays de votre employeur)*

12) Quelle est la principale nature de votre organisation ?

- Université ou organisation de recherche universitaire
- Gouvernement ou institution publique
- Organisation non-gouvernementale ou confessionnelle
- Entreprise commerciale (par ex. société pharmaceutique)
- Comité d’examen éthique
- Organisme de réglementation
- Bailleur de fonds de la recherche
- Autre

Vous avez sélectionné « Autre », veuillez préciser :

13) Quel est votre poste / titre principal ?

Plus d’informations

- Spécialiste en recherche clinique
- Statisticien / Statisticienne
- Épidémiologiste
- Gestionnaire de données
- Scientifique de données

- Bioinformaticien / Bioinformaticienne
- Professionnel(le) du soutien à la recherche
- Autre

Vous avez sélectionné « Autre », veuillez préciser :

13a) Laquelle de ces propositions vous décrit le mieux ?

Plus d’informations

- Chercheur / Chercheuse senior
- Chercheur / Chercheuse en milieu de carrière
- Chercheur / Chercheuse en début de carrière
- Étudiant(e) de troisième cycle

14) Quel est votre groupe d’âge ?

15) Quel est votre sexe ?

- Homme
- Femme
- Autre
- Je préfère ne pas répondre

16) Souhaitez-vous nous dire autre chose ?

1) Quelle est la principale raison de ne pas utiliser les données collectées par d'autres chercheurs ?

- Je n'ai pas besoin d'utiliser les données des autres pour mon travail
- Je n'ai pas trouvé de données pertinentes pour mon projet
- Je n'ai pas pu accéder aux données
- J'ai rencontré des difficultés pour utiliser les données
- Autre

Vous avez sélectionné « Autre », veuillez préciser :

1a) Des problèmes courants rencontrés lors de *l'obtention* d'ensembles de données partagés sont énumérés ci-dessous. D'après ce que vous avez pu constater, quel impact ces problèmes ont-ils eu sur votre projet ?

	Je n'ai pas rencontré ce problème	Aucun impact	Faible impact	Impact modéré	Impact élevé
Difficulté à trouver des données pertinentes					
Données non disponibles au moment de la publication des résultats de la recherche					
Processus d'accès aux données manquant de clarté					
Processus ou documentation exigé(e) trop laborieux(se)					

Données n’existant plus dans le service d’archivage des données					
Réponse lente ou inexistante du fournisseur de données					
Restrictions d’ordre éthique, légale ou privée relatives aux données					
Accès aux données refusé					
Coût des données prohibitifs					
Données fournies avec des restrictions					

Autre problème non répertorié dans le tableau ci-dessus *(veuillez préciser le problème et l’impact sur le travail que vous aviez prévu)*

1a) Des problèmes courants rencontrés lors de l’utilisation d’ensembles de données partagés sont énumérés ci-dessous. D’après ce que vous avez pu constater, quel impact ces problèmes ont-ils eu sur votre projet ?

	Je n’ai pas rencontré ce problème	Aucun impact	Faible impact	Impact modéré	Impact élevé
Les variables de données nécessaires n’étaient pas collectées dans l’ensemble de données					

Format ou structure des données inutilisable	☐	☐	☐	☐	☐
Erreurs ou incohérences dans les données	☐	☐	☐	☐	☐
Données non complètes (beaucoup de valeurs manquantes)	☐	☐	☐	☐	☐
Difficulté à comprendre les données	☐	☐	☐	☐	☐
Conception d'étude inappropriée	☐	☐	☐	☐	☐
Données insuffisantes (par ex. trop petite taille de l'échantillon)	☐	☐	☐	☐	☐
Données dans une autre langue	☐	☐	☐	☐	☐
Pas de méta données ou données limitées (dictionnaire de données, protocole, plan d'analyse statistique)	☐	☐	☐	☐	☐
Manque de ressources pour utiliser les données (par ex. analyses, logiciel, matériel, conservation des données)	☐	☐	☐	☐	☐

Autre problème non répertorié dans le tableau ci-dessus *(veuillez préciser le problème et l'impact sur le travail que vous aviez prévu)*

2) Quel genre de support ou de ressources vous permettrait d'avoir accès et d'utiliser plus efficacement les données collectées par d'autres chercheurs ?

	1 = le moins utile, 5 = le plus utile ❋ *Exigé*				
	1	2	3	4	5
Services d’archivage des données : endroit où trouver les données pertinentes	☐	☐	☐	☐	☐
Octroi de licence de données : conditions générales de l’utilisation des données	☐	☐	☐	☐	☐
Analyse : méthodes et outils appropriés pour rassembler et utiliser les données	☐	☐	☐	☐	☐
Règles en matière de recherche : comment utiliser les données de manière responsable (paternité de l’œuvre, reconnaissance d’attribution, propriété intellectuelle)	☐	☐	☐	☐	☐
Assistance légale : négociation et conclusion des accords sur le partage des données	☐	☐	☐	☐	☐
Assistance financière : lorsque les données ont un coût	☐	☐	☐	☐	☐

Autre, *veuillez préciser*

QU’EN EST-IL DE VOUS ?

3) Quelle est votre principale discipline de recherche ?

Vous avez sélectionné « Autre », veuillez préciser :

4) Dans quel pays votre employeur est-il situé ? *(Si, par exemple, vous êtes*

employé(e) par une université en Suède et que vous travaillez sur un site en Indonésie, sélectionnez Suède en tant que pays de votre employeur)

5) Quelle est la principale nature de votre organisation ?

- Université ou organisation de recherche universitaire
- Gouvernement ou institution de recherche publique
- Organisation non-gouvernementale ou confessionnelle
- Entreprise commerciale (par ex. société pharmaceutique)
- Comité d'examen éthique
- Organisme de réglementation
- Bailleur de fonds de la recherche
- Autre

Vous avez sélectionné « Autre », veuillez préciser :

6) Lequel de ces titres vous décrit le mieux ?

Plus d'informations

- Professionnel(le) non universitaire
- Chercheur / Chercheuse senior
- Chercheur / Chercheuse en milieu de carrière
- Chercheur / Chercheuse en début de carrière
- Étudiant(e) de troisième cycle
- Autre

Vous avez sélectionné « Autre », veuillez préciser :

7) Quel est votre groupe d'âge ?

8) Quel est votre sexe ?

- Homme
- Femme
- Autre
- Je préfère ne pas répondre

9) Souhaitez-vous nous dire autre chose ?

Merci d’avoir répondu à l’enquête

Si vous avez des questions concernant ce projet, veuillez envoyer un courriel à l’adresse reuse@tropmedres.ac

---

Clé pour les options de sélection

5 - 3) Combien de fois avez-vous demandé des données au cours des 5 dernières années ? (*Si vous ne vous souvenez pas du nombre exact de demandes, vous pouvez fournir une estimation*)

- 1
- 2
- 3
- 4
- 5
- Plus de 5

12- 10) Quelle est votre principale discipline de recherche ?

- Maladies infectieuses
- Santé mondiale / Santé publique
- Sciences de laboratoire clinique
- Immunologie clinique
- Microbiologie clinique
- Épidémiologie
- Génétique moléculaire
- Parasitologie
- Science dentaire
- Dermatologie
- Gynécologie
- Neurologie
- Soins infirmiers
- Histologie
- Autre

13 - 11) Dans quel pays votre employeur est-il situé ? *(Si, par exemple, vous êtes employé(e) par une université en Suède et que vous travaillez sur un site en Indonésie, sélectionnez Suède en tant que pays de votre employeur)*

Afghanistan
Afrique du Sud
Akrotiri
Albanie
Algérie
Allemagne
Andorre
Angola
Anguilla
Antarctique
Antigua-et-Barbuda
Antilles néerlandaises
Arabie saoudite
Archipel Turques-et-Caïques
Argentine
Arménie
Aruba
Australie
Autriche
Azerbaïdjan
Bahamas
Bahreïn
Bande de Gaza
Bangladesh
Barbade
Bassas da India
Belgique
Belize
Bénin
Bermudes
Bhoutan
Biélorussie
Bolivie
Bosnie-Herzégovine
Botswana
Brésil
Brunei
Bulgarie
Burkina Faso
Burundi

Cambodge
Cameroun
Canada
Cap-Vert
Chili
Chine
Chypre
Cisjordanie
Colombie
Comores
Corée du Nord
Corée du Sud
Costa Rica
Côte d’Ivoire
Croatie
Cuba
Danemark
Dhekelia
Djibouti
Dominique
Égypte
El Salvador
Émirats arabes unis
Équateur
Érythrée
Espagne
Estonie
États fédérés de Micronésie
États-Unis
Éthiopie
Fidji
Finlande
France
Gabon
Gambie
Géorgie
Géorgie du Sud-et-les îles Sandwich du Sud
Ghana
Gibraltar
Grèce

Grenade
Groenland
Guadeloupe
Guam
Guatemala
Guernesey
Guinée
Guinée équatoriale
Guinée-Bissau
Guyane
Guyane française
Haïti
Honduras
Hong Kong
Hongrie
île Bouvet
Île Christmas
Île de la Navasse
Île de la Réunion
Île de Man
Île Europa
Île Juan de Nova
Île Norfolk
Île Tromelin
Île Wake
Îles Ashmore et Cartier
Îles Caïmans
Îles Cocos (Keeling)
Îles Cook
Îles de la mer de Corail
Îles Féroé
îles Glorieuses
Îles Heard-et-MacDonald
Îles Malouines (Îles Falkland)
Îles Mariannes du Nord
Îles Marshall
Îles Paracels
Îles Pitcairn
Îles Salomon
Îles Spratleys

Îles Vierges
Îles Vierges britanniques
Îlot de Clipperton
Inde
Indonésie
Irak
Iran
Irlande
Islande
Israël
Italie
Jamaïque
Jan Mayen
Japon
Jersey
Jordanie
Kazakhstan
Kenya
Kirghizistan
Kiribati
Koweït
Laos
Lesotho
Lettonie
Liban
Liberia
Libye
Liechtenstein
Lituanie
Luxembourg
Macao
Macédoine
Madagascar
Malaisie
Malawi
Maldives
Mali
Malte
Maroc
Martinique

Maurice
Mauritanie
Mayotte
Mexique
Moldavie
Monaco
Mongolie
Monténégro
Montserrat
Mozambique
Myanmar
Namibie
Nauru
Népal
Nicaragua
Niger
Nigeria
Niué
Norvège
Nouvelle Calédonie
Nouvelle-Zélande
Oman
Ouganda
Ouzbékistan
Pakistan
Palaos
Panama
Papouasie-Nouvelle-Guinée
Paraguay
Pays-Bas
Pérou
Philippines
Pologne
Polynésie française
Porto Rico
Portugal
Qatar
République centrafricaine
République démocratique du Congo
République Dominicaine

République du Congo
République tchèque
Roumanie
Royaume-Uni
Russie
Rwanda
Sahara occidental
Saint-Christophe-et-Niévès
Sainte Lucie
Sainte-Hélène
Saint-Marin
Saint-Pierre-et-Miquelon
Saint-Siège (Cité du Vatican)
Saint-Vincent-et-les-Grenadines
Samoa
Samoa américaine
Sao Tomé-et-Principe
Sénégal
Serbie
Seychelles
Sierra Leone
Singapour
Slovaquie
Slovénie
Somalie
Soudan
Sri Lanka
Suède
Suisse
Suriname
Svalbard
Swaziland
Syrie
Tadjikistan
Taïwan
Tanzanie
Tchad
Terres australes et antarctiques françaises
Territoire britannique de l’océan Indien
Thaïlande

Timor oriental
Togo
Tokelau
Tonga
Trinité-et-Tobago
Tunisie
Turkménistan
Turquie
Tuvalu
Ukraine
Uruguay
Vanuatu
Venezuela
Vietnam
Wallis-et-Futuna
Yémen
Zambie
Zimbabwe

16 - 14) Quel est votre groupe d’âge ?

18-24 ans
25-34 ans
35-44 ans
45-54 ans
55-64 ans
65-74 ans
75 ans ou plus
Je préfère ne pas répondre

21 - 3) Quelle est votre principale discipline de recherche ?

Maladies infectieuses
Santé mondiale / Santé publique
Sciences de laboratoire clinique
Immunologie clinique
Microbiologie clinique
Épidémiologie
Génétique moléculaire
Parasitologie
Science dentaire

Dermatologie
Gynécologie
Neurologie
Soins infirmiers
Histologie
Autre

22 - 4) Dans quel pays votre employeur est-il situé ? ***(Si, par exemple, vous êtes employé(e) par une université en Suède et que vous travaillez sur un site en Indonésie, sélectionnez Suède en tant que pays de votre employeur)***

Afghanistan
Afrique du Sud
Akrotiri
Albanie
Algérie
Allemagne
Andorre
Angola
Anguilla
Antarctique
Antigua-et-Barbuda
Antilles néerlandaises
Arabie saoudite
Archipel Turques-et-Caïques
Argentine
Arménie
Aruba
Australie
Autriche
Azerbaïdjan
Bahamas
Bahreïn
Bande de Gaza
Bangladesh
Barbade
Bassas da India
Belgique
Belize
Bénin
Bermudes

Bhoutan
Biélorussie
Bolivie
Bosnie-Herzégovine
Botswana
Brésil
Brunei
Bulgarie
Burkina Faso
Burundi
Cambodge
Cameroun
Canada
Cap-Vert
Chili
Chine
Chypre
Cisjordanie
Colombie
Comores
Corée du Nord
Corée du Sud
Costa Rica
Côte d’Ivoire
Croatie
Cuba
Danemark
Dhekelia
Djibouti
Dominique
Égypte
El Salvador
Émirats arabes unis
Équateur
Érythrée
Espagne
Estonie
États fédérés de Micronésie
États-Unis
Éthiopie

Fidji
Finlande
France
Gabon
Gambie
Géorgie
Géorgie du Sud-et-les îles Sandwich du Sud
Ghana
Gibraltar
Grèce
Grenade
Groenland
Guadeloupe
Guam
Guatemala
Guernesey
Guinée
Guinée équatoriale
Guinée-Bissau
Guyane
Guyane française
Haïti
Honduras
Hong Kong
Hongrie
île Bouvet
Île Christmas
Île de la Navasse
Île de la Réunion
Île de Man
Île Europa
Île Juan de Nova
Île Norfolk
Île Tromelin
Île Wake
Îles Ashmore et Cartier
Îles Caïmans
Îles Cocos (Keeling)
Îles Cook
Îles de la mer de Corail

Îles Féroé
îles Glorieuses
Îles Heard-et-MacDonald
Îles Malouines (Îles Falkland)
Îles Mariannes du Nord
Îles Marshall
Îles Paracels
Îles Pitcairn
Îles Salomon
Îles Spratleys
Îles Vierges
Îles Vierges britanniques
Îlot de Clipperton
Inde
Indonésie
Irak
Iran
Irlande
Islande
Israël
Italie
Jamaïque
Jan Mayen
Japon
Jersey
Jordanie
Kazakhstan
Kenya
Kirghizistan
Kiribati
Koweït
Laos
Lesotho
Lettonie
Liban
Liberia
Libye
Liechtenstein
Lituanie
Luxembourg

Macao
Macédoine
Madagascar
Malaisie
Malawi
Maldives
Mali
Malte
Maroc
Martinique
Maurice
Mauritanie
Mayotte
Mexique
Moldavie
Monaco
Mongolie
Monténégro
Montserrat
Mozambique
Myanmar
Namibie
Nauru
Népal
Nicaragua
Niger
Nigeria
Niué
Norvège
Nouvelle Calédonie
Nouvelle-Zélande
Oman
Ouganda
Ouzbékistan
Pakistan
Palaos
Panama
Papouasie-Nouvelle-Guinée
Paraguay
Pays-Bas

Pérou
Philippines
Pologne
Polynésie française
Porto Rico
Portugal
Qatar
République centrafricaine
République démocratique du Congo
République Dominicaine
République du Congo
République tchèque
Roumanie
Royaume-Uni
Russie
Rwanda
Sahara occidental
Saint-Christophe-et-Niévès
Sainte Lucie
Sainte-Hélène
Saint-Marin
Saint-Pierre-et-Miquelon
Saint-Siège (Cité du Vatican)
Saint-Vincent-et-les-Grenadines
Samoa
Samoa américaine
Sao Tomé-et-Principe
Sénégal
Serbie
Seychelles
Sierra Leone
Singapour
Slovaquie
Slovénie
Somalie
Soudan
Sri Lanka
Suède
Suisse
Suriname

Svalbard
Swaziland
Syrie
Tadjikistan
Taïwan
Tanzanie
Tchad
Terres australes et antarctiques françaises
Territoire britannique de l’océan Indien
Thaïlande
Timor oriental
Togo
Tokelau
Tonga
Trinité-et-Tobago
Tunisie
Turkménistan
Turquie
Tuvalu
Ukraine
Uruguay
Vanuatu
Venezuela
Vietnam
Wallis-et-Futuna
Yémen
Zambie
Zimbabwe

25 - 7) Quel est votre groupe d’âge ?

18-24 ans
25-34 ans
35-44 ans
45-54 ans
55-64 ans
65-74 ans
75 ans ou plus
Je préfère ne pas répondre
