# Supplementary material for: Investigating the Secondary Use of Clinical Research Data: Protocol for a Mixed Methods Study
Source: JMIR Res Protoc. 2023 Mar 6;12:e44875. doi: 10.2196/44875 (PMC10028503; doi:10.2196/44875)
Supplement: Multimedia Appendix 4 [file resprot_v12i1e44875_app4.pdf]

Este questionário faz parte de um estudo realizado por investigadores da Universidade de Oxford para compreender se e como os investigadores médicos utilizam dados recolhidos por outros. Mesmo que não utilize dados recolhidos por outros, o seu conhecimento é útil para este estudo. As suas respostas são anónimas. O questionário demora **3 -7** minutos a preencher.

Qual é o objetivo da investigação?

Está a tornar-se cada vez mais a norma partilhar dados de estudos de investigação clínica. Procuramos compreender se e como os conjuntos de dados partilhados são reutilizados, que desafios existem no acesso e reutilização dos dados, e qual o impacto da reutilização de dados na investigação científica e na saúde pública geral.

Quais são os possíveis benefícios desta investigação?

Os dados deste estudo vão ajudar a definir quais as medidas a implementar para acelerar efetivamente a utilização de dados secundários. Antecipamos que o aumento da reutilização de dados resultará numa melhoria da qualidade e da transparência da ciência, da saúde pública e dos resultados dos pacientes, e num melhor retorno do investimento em investigação.

Informações e equipa de estudo

O estudo é conduzido por investigadores da Universidade de Oxford e da Unidade de Investigação de Medicina Tropical Mahidol Oxford com colaborações no Reino Unido, no Quénia e no Vietname. O estudo foi aprovado pela Comissão de Ética de Investigação Tropical de Oxford (OxTREC), número de referência: 568-20.

Proteção de dados

No preenchimento de um questionário, irá fornecer informação sobre si mesmo/a ("dados pessoais"). Os regulamentos de proteção de dados requerem que definamos a base legal para o processamento de informações sobre si. No caso de investigação, trata-se de "uma tarefa do interesse público". A Universidade de Oxford é o controlador de dados e é responsável pela curadoria das suas informações e por utilizá-las em conformidade com o Regulamento Geral de Proteção de Dados e legislação de proteção de dados associada. Os seus dados serão conservados em segurança, em conformidade com as políticas e procedimentos da Universidade. Estão disponíveis mais informações no website de segurança das informações da Universidade. As informações sobre os seus direitos relativamente aos dados pessoais são explicadas aqui.

Os resultados deste projeto de investigação serão disseminados através de relatórios de investigação, publicações/artigos e apresentações. Processamos os seus dados para esse efeito apenas porque recebemos o seu consentimento nesse sentido, assinalando a caixa apropriada. As suas respostas serão anónimas, o questionário não recolhe o seu nome, endereço de e-mail ou endereço IP. Como tal, após enviá-las, não poderemos retirar as suas respostas retroativamente. Se sair do questionário antes de submeter as respostas, os seus dados não serão guardados.

Contacto

Se pretender apresentar dúvidas ou preocupações sobre a utilização que fazemos dos seus dados, por favor, contacte-nos através do e-mail *reuse@tropmedres.ac* ou por telefone +66 02 203 6333 Ext 8302
Se pretender contactar alguém independente da equipa de estudo, pode contactar a Comissão de Ética de Investigação Tropical de Oxford (OxTREC) por e-mail: oxtrec@admin.ox.ac.uk.

Proteção de dados

Mais informações

Concordo em participar no questionário. Compreendo que a minha participação é voluntária e que tenho a liberdade de sair a qualquer altura (por favor, marque)

Já utilizou dados de investigação clínica que tenham sido partilhados por outros investigadores?

- ○ Sim
- ○ Não

1) Que tipo de dados, partilhados por outros investigadores, já utilizou? *(marque todas as respostas aplicáveis)*

- ☐ Dados de ensaios clínicos
- ☐ Inquéritos de saúde transversais
- ☐ Dados de coorte observacionais
- ☐ Dados de vigilância
- ☐ Dados administrativos
- ☐ Registos de paciente/doença
- ☐ Dados farmacológicos
- ☐ Dados de biologia molecular
- ☐ Dados ómicos (Genómica, Transcriptómica, Proteómica, Epigenómica, Metabolómica)
- ☐ Dados de ciências sociais
- ☐ Dados de economia da saúde
- ☐ Dados qualitativos
- ☐ Outros

Selecionou dados Qualitativos ou "Outros". Especifique que tipo de investigação gerou os dados

2) Como acedeu aos conjuntos de dados? *(marque todas as respostas aplicáveis)*

- ☐ Pediu à entidade de recolha/conservação dos dados
- ☐ Transferiu de um website/repositório público
- ☐ Solicitou através de uma Comissão de Acesso a Dados
- ☐ Outro

Se selecionou Outro, por favor, especifique:

3) Quantas vezes solicitou dados nos últimos 5 anos? *(Se não se lembrar do número exato de pedidos, pode fornecer uma estimativa)*

Em 2017

Em 2018

Em 2019

Em 2020

Em 2021

4) Para que utilizou os dados? *(marque todas as respostas aplicáveis)*

- ☐ Planear e projetar um novo estudo (por ex.: determinar a exequibilidade de nova investigação)
- ☐ Compilados ou meta-análises
- ☐ Modelagem matemática
- ☐ Treino de algoritmos de inteligência artificial
- ☐ Reanálise para verificar resultados da investigação
- ☐ Análise secundária (incluindo geração/teste de novas hipóteses)
- ☐ Dados de referência para um novo estudo
- ☐ Ensino
- ☐ Análise de literatura
- ☐ Candidatura ao registo de um medicamento/dispositivo médico
- ☐ Desenvolvimento de uma diretriz/política de âmbito sanitário
- ☐ Outro

Se selecionou Outro, por favor, especifique:

____

5) O que geraram os dados? *(selecione todas as categorias adequadas)*

- ☐ Publicação
- ☐ Apresentação (por ex.: palestra, póster, seminário, *webinar*)
- ☐ Tese ou Dissertação
- ☐ Relatório
- ☐ Capítulo de um livro
- ☐ Validação de modelo matemático
- ☐ Treino de algoritmos de inteligência artificial
- ☐ Validação de código de software

- [ ] Publicação num blogue
- [ ] Publicação em redes sociais
- [ ] Outro
- [ ] Nada foi gerado da utilização dos dados

Se selecionou Outro, por favor, especifique:

Indique o número total de ***Publicações*** com base nos dados.

- 1-4
- 5-10
- Mais de 10

Indique o número total de ***Apresentações*** com base nos dados.

- 1-4
- 5-10
- Mais de 10

Indique o número total de ***Teses ou Dissertações*** obtido a partir da utilização dos dados.

- 1-4
- 5-10
- Mais de 10

Indique o número total de ***Relatórios*** gerados.

- 1-4
- 5-10

- Mais de 10

Indique o número total de ***Capítulos de um livro*** com base nos dados.

- 1-4
- 5-10
- Mais de 10

Indique o número total de ***modelos matemáticos*** *validados*.

- 1-4
- 5-10
- Mais de 10

Indique o número total de ***algoritmos de inteligência artificial*** treinados.

- 1-4
- 5-10
- Mais de 10

Indique o número total de ***fragmentos de código de software*** validado.

- 1-4
- 5-10
- Mais de 10

Indique o número total de ***Publicações num blogue*** com base nos dados.

- 1-4
- 5-10
- Mais de 10

Indique o número total de ***Publicações em redes sociais*** com base nos dados.

- 1-4
- 5-10
- Mais de 10

Indique o número total de ***produtos mencionados em “Outros”***.

- 1-4
- 5-10
- Mais de 10

6) Quais foram os resultados da utilização de dados? *(marque todas as respostas aplicáveis)*

- Resultados validados ou verificados a partir de publicações existentes
- Correção ou retratação de uma publicação existente
- Obtenção de um Mestrado ou Doutoramento
- Criação de novo posto, por ex.: internato, contratação de novo analista
- Progressão na carreira para mim ou os meus pares
- Aumento de citações e de visibilidade para mim/a minha instituição
- Lançamento de estudos com dados secundários como referência
- Projeção de um novo estudo influenciada pelos resultados da análise
- Colaboração nova/potenciada com outros investigadores
- Convite para discursar/contribuir num fórum de especialistas
- Menção por um organismo autorizado, por ex.: órgão governamental, OMS
- Resultados incluídos em políticas de saúde/diretrizes de tratamento
- Registo de um novo medicamento ou dispositivo médico
- Financiamento da minha investigação ou instituição

- [ ] Vantagem financeira do registo de um medicamento ou dispositivo médico
- [ ] Vantagem financeira pessoal, por ex.: pagamento por consultadoria para análise secundária
- [ ] Outros
- [ ] Nenhum

Se selecionou Outros, por favor, especifique:

7) Encontrará abaixo alguns problemas comuns sentidos ao *obter* conjuntos de dados partilhados. Na sua experiência, que impacto tiveram esses problemas no seu trabalho planeado?

	✳ *Obrigatório*				
	Não me deparei com este problema	Nenhum impacto	Pouco impacto	Impacto moderado	Grande impacto
Dificuldade em encontrar dados relevantes	☐	☐	☐	☐	☐
Dados indisponíveis aquando da publicação dos resultados da investigação	☐	☐	☐	☐	☐
Processo pouco claro para aceder aos dados	☐	☐	☐	☐	☐
Processo ou documentação exigida demasiado complicados	☐	☐	☐	☐	☐
Os dados já não existem no repositório	☐	☐	☐	☐	☐
Pouca ou nenhuma resposta do fornecedor de dados	☐	☐	☐	☐	☐
Restrições éticas, legais ou de privacidade relativamente aos dados	☐	☐	☐	☐	☐
Acesso negado aos dados	☐	☐	☐	☐	☐
Custo dos dados proibitivo	☐	☐	☐	☐	☐
Os dados foram fornecidos sem restrições	☐	☐	☐	☐	☐

Outros problemas não listados na tabela acima. *(Por favor, especifique o problema e o impacto no seu trabalho planeado)*

8) Encontrará abaixo algumas dificuldades comuns sentidas ao *utilizar* conjuntos de dados partilhados. Na sua experiência, que impacto tiveram esses problemas no seu trabalho planeado?

	✳ *Obrigatório*				
	Não me deparei com este problema	Nenhum impacto	Pouco impacto	Impacto moderado	Grande impacto
As variáveis dos dados necessárias não foram compiladas no conjunto de dados	☐	☐	☐	☐	☐
Estrutura ou formato de dados inutilizável	☐	☐	☐	☐	☐
Erros ou inconsistências nos dados	☐	☐	☐	☐	☐
Incompletude de dados (muitos valores em falta)	☐	☐	☐	☐	☐
Dificuldade na compreensão dos dados	☐	☐	☐	☐	☐
Elaboração inadequada do estudo	☐	☐	☐	☐	☐
Dados insuficientes (por ex.: tamanho da amostra demasiado pequeno)	☐	☐	☐	☐	☐
Os dados estavam num idioma diferente	☐	☐	☐	☐	☐
Metadados limitados ou ausentes (dicionário de dados, protocolo, plano de análise estatística)	☐	☐	☐	☐	☐
Falta de recursos para utilizar os dados (por ex.: análise, software, hardware, curadoria de dados)	☐	☐	☐	☐	☐

Outros problemas não listados na tabela acima. *(Por favor, especifique o problema e o impacto no seu trabalho planeado)*

9) Que tipo de apoio ou recursos lhe permitiriam aceder e utilizar os dados recolhidos por outros investigadores mais eficazmente?

	1=menos útil, 5=mais útil				
	1	2	3	4	5
Repositórios: onde encontrar dados relevantes	☐	☐	☐	☐	☐
Licenciamento de dados: termos e condições para a utilização de dados	☐	☐	☐	☐	☐
Análise: ferramentas e métodos apropriados para reunir e utilizar dados	☐	☐	☐	☐	☐
Etiqueta de investigação: como utilizar dados responsavelmente (autoria, atribuição de reconhecimento, propriedade intelectual)	☐	☐	☐	☐	☐
Assistência jurídica: negociação e execução de acordos de partilha de dados	☐	☐	☐	☐	☐
Assistência financeira: quando os dados têm um custo	☐	☐	☐	☐	☐

Outro, *por favor, especifique*

ACERCA DE SI

10) Qual é a sua principal disciplina de investigação?

Se selecionou Outros, por favor, especifique:

11) Em que país se encontra a sua entidade empregadora? *(se, por exemplo, for funcionário de uma universidade da Suécia e trabalhar no terreno na Indonésia, selecione Suécia como o país do seu empregador)*

12) Qual é a principal natureza da sua organização?

- Universidade ou organização de investigação académica
- Instituição pública ou governamental
- Organização não governamental ou confessional
- Organização comercial (por ex.: empresa farmacêutica)
- Comissão de Ética
- Autoridade reguladora
- Financiador da investigação
- Outra

Se selecionou Outra, por favor, especifique:

13) Qual é o seu principal cargo/função?

Mais informações

- Investigador clínico
- Estatístico
- Epidemiologista
- Gestor de dados
- Cientista de dados

- Bioinformático
- Profissional de apoio à investigação
- Outro

Se selecionou Outro, por favor, especifique:

13a) Qual dos seguintes melhor o/a descreve?

Mais informações

- Investigador sénior
- Investigador intermédio
- Investigador em início de carreira
- Aluno licenciado

14) Qual é a sua faixa etária?

15) Qual é o seu género?

- Masculino
- Feminino
- Outro
- Prefiro não dizer

16) Há algo mais que gostaria de nos informar?

1) Qual é o principal motivo para não utilizar dados recolhidos por outros investigadores?

- Não preciso de utilizar dados de outros para o meu trabalho
- Não encontrei dados relevantes para o meu projeto
- Não consegui aceder aos dados
- Tive dificuldade em utilizar os dados
- Outro

Se selecionou Outro, por favor, especifique:

1a) Encontrará abaixo alguns problemas comuns sentidos ao *obter* conjuntos de dados partilhados. Na sua experiência, que impacto tiveram esses problemas no seu projeto?

	Não me deparei com este problema	Nenhum impacto	Pouco impacto	Impacto moderado	Grande impacto
Dificuldade em encontrar dados relevantes					
Dados indisponíveis aquando da publicação dos resultados da investigação					
Processo pouco claro para aceder aos dados					
Processo ou documentação exigida demasiado complicados					

Os dados já não existem no repositório	☐	☐	☐	☐	☐
Pouca ou nenhuma resposta do fornecedor de dados	☐	☐	☐	☐	☐
Restrições éticas, legais ou de privacidade relativamente aos dados	☐	☐	☐	☐	☐
Acesso negado aos dados	☐	☐	☐	☐	☐
Custo dos dados proibitivo	☐	☐	☐	☐	☐
Os dados foram fornecidos sem restrições	☐	☐	☐	☐	☐

Outro problema não listado na tabela acima *(por favor, especifique o problema e o impacto no seu trabalho planeado)*

1a) Encontrará abaixo algumas dificuldades comuns sentidas ao utilizar conjuntos de dados partilhados. Na sua experiência, que impacto tiveram esses problemas no seu projeto?

	Não me deparei com este problema	Nenhum impacto	Pouco impacto	Impacto moderado	Grande impacto
As variáveis dos dados necessárias não foram compiladas no conjunto de dados	☐	☐	☐	☐	☐

Estrutura ou formato de dados inutilizável	☐	☐	☐	☐	☐
Erros ou inconsistências nos dados	☐	☐	☐	☐	☐
Incompletude de dados (muitos valores em falta)	☐	☐	☐	☐	☐
Dificuldade na compreensão dos dados	☐	☐	☐	☐	☐
Elaboração inadequada do estudo	☐	☐	☐	☐	☐
Dados insuficientes (por ex.: tamanho da amostra demasiado pequeno)	☐	☐	☐	☐	☐
Os dados estavam num idioma diferente	☐	☐	☐	☐	☐
Metadados limitados ou ausentes (dicionário de dados, protocolo, plano de análise estatística)	☐	☐	☐	☐	☐
Falta de recursos para utilizar os dados (por ex.: análise, software, hardware, curadoria de dados)	☐	☐	☐	☐	☐

Outro problema não listado na tabela acima *(por favor, especifique o problema e o impacto no seu trabalho planeado)*

2) Que tipo de apoio ou recursos lhe permitiriam aceder e utilizar os dados recolhidos por outros investigadores mais eficazmente?

	**1=menos útil, 5=mais útil** ✳ *Obrigatório*				
	1	2	3	4	5
Repositórios: onde encontrar dados relevantes	☐	☐	☐	☐	☐
Licenciamento de dados: termos e condições para a utilização de dados	☐	☐	☐	☐	☐
Análise: ferramentas e métodos apropriados para reunir e utilizar os dados	☐	☐	☐	☐	☐
Etiqueta de investigação: como utilizar dados responsavelmente (autoria, atribuição de reconhecimento, propriedade intelectual)	☐	☐	☐	☐	☐
Assistência jurídica: negociação e execução de acordos de partilha de dados	☐	☐	☐	☐	☐
Assistência financeira: quando os dados têm um custo	☐	☐	☐	☐	☐

Outro, *por favor, especifique*

ACERCA DE SI

3) Qual é a sua principal disciplina de investigação?

Se selecionou Outra, por favor, especifique:

4) Em que país se encontra a sua entidade empregadora? *(se, por exemplo, for funcionário de uma universidade da Suécia e trabalhar no terreno na Indonésia, selecione Suécia como o país do seu empregador)*

5) Qual é a principal natureza da sua organização?

- Universidade ou organização de investigação académica
- Instituição de investigação pública ou governamental
- Organização não governamental ou confessional
- Organização comercial (por ex.: empresa farmacêutica)
- Comissão de Ética
- Autoridade reguladora
- Financiador da investigação
- Outra

Se selecionou Outra, por favor, especifique:

6) Qual dos seguintes títulos melhor o/a descreve?

Mais informações

- Profissional não académico
- Investigador sénior
- Investigador intermédio

- Investigador em início de carreira
- Aluno licenciado
- Outro

Se selecionou Outro, por favor, especifique:

7) Qual é a sua faixa etária?

8) Qual é o seu género?

- Masculino
- Feminino
- Outro
- Prefiro não dizer

9) Há algo mais que gostaria de nos informar?

Obrigado por ter completado o questionário

Se tiver alguma questão sobre este projeto, envie um e-mail para reuse@tropmedres.ac

---

Importante para as opções de seleção

5 - 3) Quantas vezes solicitou dados nos últimos 5 anos? (*se não se lembrar do número exato de pedidos, pode fornecer uma estimativa*)

1
2
3
4
5
Mais de 5

12 - 10) Qual é a sua principal disciplina de investigação?

Doenças infeciosas
Saúde pública/global
Ciências laboratoriais clínicas
Imunologia clínica
Microbiologia clínica
Epidemiologia
Genética molecular
Parasitologia
Ciência dentária
Dermatologia
Ginecologia
Neurologia
Enfermagem
Histologia
Outra

13 - 11) Em que país se encontra a sua entidade empregadora? (*se, por exemplo, for funcionário de uma universidade da Suécia e trabalhar no terreno na Indonésia, selecione Suécia como o país do seu empregador*)

Afeganistão

Acrotíri
Albânia
Argélia
Samoa Americana
Andorra
Angola
Anguila
Antártida
Antígua e Barbuda
Argentina
Arménia
Aruba
Ilhas Ashmore e Cartier
Austrália
Áustria
Azerbaijão
Bahamas
Barém
Bangladeche
Barbados
Bassas da Índia
Bielorrússia
Bélgica
Belize
Benim
Bermudas
Butão
Bolívia
Bósnia e Herzegovina
Botsuana
Ilha Bouvet
Brasil
Território Britânico do Oceano Índico
Ilhas Virgens Britânicas
Brunei
Bulgária
Burquina Faso
Myanmar
Burundi
Camboja

Camarões
Canadá
Cabo Verde
Ilhas Caimão
República Centro-Africana
Chade
Chile
China
Ilha do Natal
Ilha de Clipperton
Ilhas Cocos (Keeling)
Colômbia
Comores
Congo, República Democrática do
Congo, República do
Ilhas Cook
Ilhas do Mar de Coral
Costa Rica
Costa do Marfim
Croácia
Cuba
Chipre
Chéquia
Dinamarca
Deceleia
Djibuti
Dominica
República Dominicana
Equador
Egito
El Salvador
Guiné Equatorial
Eritreia
Estónia
Etiópia
Ilha Europa
Ilhas Falkland (Ilhas Malvinas)
Ilhas Faroé
Fiji
Finlândia

França
Guiana Francesa
Polinésia Francesa
Terras Austrais e Antárticas Francesas
Gabão
Gâmbia
Faixa de Gaza
Geórgia
Alemanha
Gana
Gibraltar
Ilhas Gloriosas
Grécia
Gronelândia
Granada
Guadalupe
Guam
Guatemala
Guernsey
Guiné
Guiné-Bissau
Guiana
Haiti
Ilhas Heard e McDonald
Santa Sé (Cidade do Vaticano)
Honduras
Hong Kong
Hungria
Islândia
Índia
Indonésia
Irão
Iraque
Irlanda
Ilha de Man
Israel
Itália
Jamaica
Jan Mayen
Japão

Jersey
Jordânia
Ilha Juan de Nova
Cazaquistão
Quénia
Quiribati
Coreia do Norte
Coreia do Sul
Kuwait
Quirguistão
Laos
Letónia
Líbano
Lesoto
Libéria
Líbia
Liechtenstein
Lituânia
Luxemburgo
Macau
Macedónia
Madagáscar
Maláui
Malásia
Maldivas
Mali
Malta
Ilhas Marshall
Martinica
Mauritânia
Maurícia
Maiote
México
Micronésia, Estados Federados da
Moldávia
Mónaco
Mongólia
Montenegro
Monserrate
Marrocos

Moçambique
Namíbia
Nauru
Ilha de Navassa
Nepal
Holanda
Antilhas Holandesas
Nova Caledónia
Nova Zelândia
Nicarágua
Níger
Nigéria
Niue
Ilha Norfolk
Ilhas Marianas Setentrionais
Noruega
Omã
Paquistão
Palau
Panamá
Papua-Nova Guiné
Ilhas Paracel
Paraguai
Peru
Filipinas
Ilhas Pitcairn
Polónia
Portugal
Porto Rico
Catar
Reunião
Roménia
Rússia
Ruanda
Santa Helena
São Cristóvão e Nevis
Santa Lúcia
São Pedro e Miquelon
São Vicente e as Granadinas
Samoa

San Marino
São Tomé e Príncipe
Arábia Saudita
Senegal
Sérvia
Seicheles
Serra Leoa
Singapura
Eslováquia
Eslovénia
Ilhas Salomão
Somália
África do Sul
Ilhas de Geórgia do Sul e Sandwich do Sul
Espanha
Ilhas Spratly
Sri Lanka
Sudão
Suriname
Svalbard
Suazilândia
Suécia
Suíça
Síria
Taiwan
Tajiquistão
Tanzânia
Tailândia
Timor-Leste
Togo
Toquelau
Tonga
Trinidad e Tobago
Ilha Tromelin
Tunísia
Turquia
Turquemenistão
Ilhas Turcas e Caicos
Tuvalu
Uganda

Ucrânia
Emirados Árabes Unidos
Reino Unido
Estados Unidos
Uruguai
Uzbequistão
Vanuatu
Venezuela
Vietname
Ilhas Virgens
Ilha Wake
Wallis e Futuna
Cisjordânia
Sara Ocidental
Iémen
Zâmbia
Zimbábue

16 - 14) Qual é a sua faixa etária?

18-24
25-34
35-44
45-54
55-64
65-74
75 ou mais
Prefiro não dizer

21 - 3) Qual é a sua principal disciplina de investigação?

Doenças infeciosas
Saúde pública/global
Ciências laboratoriais clínicas
Imunologia clínica
Microbiologia clínica
Epidemiologia
Genética molecular
Parasitologia
Ciência dentária
Dermatologia

Ginecologia
Neurologia
Enfermagem
Histologia
Outra

22 - 4) Em que país se encontra a sua entidade empregadora? ***(se, por exemplo, for funcionário de uma universidade da Suécia e trabalhar no terreno na Indonésia, selecione Suécia como o país do seu empregador)***

Afeganistão
Acrotíri
Albânia
Argélia
Samoa Americana
Andorra
Angola
Anguila
Antártida
Antígua e Barbuda
Argentina
Arménia
Aruba
Ilhas Ashmore e Cartier
Austrália
Áustria
Azerbaijão
Bahamas
Barém
Bangladeche
Barbados
Bassas da Índia
Bielorrússia
Bélgica
Belize
Benim
Bermudas
Butão
Bolívia
Bósnia e Herzegovina
Botsuana

Ilha Bouvet
Brasil
Território Britânico do Oceano Índico
Ilhas Virgens Britânicas
Brunei
Bulgária
Burquina Faso
Myanmar
Burundi
Camboja
Camarões
Canadá
Cabo Verde
Ilhas Caimão
República Centro-Africana
Chade
Chile
China
Ilha do Natal
Ilha de Clipperton
Ilhas Cocos (Keeling)
Colômbia
Comores
Congo, República Democrática do
Congo, República do
Ilhas Cook
Ilhas do Mar de Coral
Costa Rica
Costa do Marfim
Croácia
Cuba
Chipre
Chéquia
Dinamarca
Deceleia
Djibuti
Dominica
República Dominicana
Equador
Egito

El Salvador
Guiné Equatorial
Eritreia
Estónia
Etiópia
Ilha Europa
Ilhas Falkland (Ilhas Malvinas)
Ilhas Faroé
Fiji
Finlândia
França
Guiana Francesa
Polinésia Francesa
Terras Austrais e Antárticas Francesas
Gabão
Gâmbia
Faixa de Gaza
Geórgia
Alemanha
Gana
Gibraltar
Ilhas Gloriosas
Grécia
Gronelândia
Granada
Guadalupe
Guam
Guatemala
Guernsey
Guiné
Guiné-Bissau
Guiana
Haiti
Ilhas Heard e McDonald
Santa Sé (Cidade do Vaticano)
Honduras
Hong Kong
Hungria
Islândia
Índia

Indonésia
Irão
Iraque
Irlanda
Ilha de Man
Israel
Itália
Jamaica
Jan Mayen
Japão
Jersey
Jordânia
Ilha Juan de Nova
Cazaquistão
Quénia
Quiribati
Coreia do Norte
Coreia do Sul
Kuwait
Quirguistão
Laos
Letónia
Líbano
Lesoto
Libéria
Líbia
Liechtenstein
Lituânia
Luxemburgo
Macau
Macedónia
Madagáscar
Maláui
Malásia
Maldivas
Mali
Malta
Ilhas Marshall
Martinica
Mauritânia

Maurícia
Maiote
México
Micronésia, Estados Federados da
Moldávia
Mónaco
Mongólia
Montenegro
Monserrate
Marrocos
Moçambique
Namíbia
Nauru
Ilha de Navassa
Nepal
Holanda
Antilhas Holandesas
Nova Caledónia
Nova Zelândia
Nicarágua
Níger
Nigéria
Niue
Ilha Norfolk
Ilhas Marianas Setentrionais
Noruega
Omã
Paquistão
Palau
Panamá
Papua-Nova Guiné
Ilhas Paracel
Paraguai
Peru
Filipinas
Ilhas Pitcairn
Polónia
Portugal
Porto Rico
Catar

Reunião
Roménia
Rússia
Ruanda
Santa Helena
São Cristóvão e Nevis
Santa Lúcia
São Pedro e Miquelon
São Vicente e as Granadinas
Samoa
San Marino
São Tomé e Príncipe
Arábia Saudita
Senegal
Sérvia
Seicheles
Serra Leoa
Singapura
Eslováquia
Eslovénia
Ilhas Salomão
Somália
África do Sul
Ilhas de Geórgia do Sul e Sandwich do Sul
Espanha
Ilhas Spratly
Sri Lanka
Sudão
Suriname
Svalbard
Suazilândia
Suécia
Suíça
Síria
Taiwan
Tajiquistão
Tanzânia
Tailândia
Timor-Leste
Togo

Toquelau
Tonga
Trinidad e Tobago
Ilha Tromelin
Tunísia
Turquia
Turquemenistão
Ilhas Turcas e Caicos
Tuvalu
Uganda
Ucrânia
Emirados Árabes Unidos
Reino Unido
Estados Unidos
Uruguai
Uzbequistão
Vanuatu
Venezuela
Vietname
Ilhas Virgens
Ilha Wake
Wallis e Futuna
Cisjordânia
Sara Ocidental
Iémen
Zâmbia
Zimbábue

25 - 7) Qual é a sua faixa etária?

18-24
25-34
35-44
45-54
55-64
65-74
75 ou mais
Prefiro não dizer
