# Supplementary material for: Investigating the Secondary Use of Clinical Research Data: Protocol for a Mixed Methods Study
Source: JMIR Res Protoc. 2023 Mar 6;12:e44875. doi: 10.2196/44875 (PMC10028503; doi:10.2196/44875)
Supplement: Multimedia Appendix 5 [file resprot_v12i1e44875_app5.pdf]

Tái sử dụng dữ liệu nghiên cứu lâm sàng

Cuộc khảo sát tự nguyện này là một phần của nghiên cứu do các nhà nghiên cứu từ Đại học Oxford chủ trì nhằm tìm hiểu khả năng và cách các nhà nghiên cứu y tế sử dụng dữ liệu do những người khác thu thập. Ngay cả khi bạn không sử dụng dữ liệu do người khác thu thập, những hiểu biết của bạn cũng rất hữu ích cho nghiên cứu này. Các câu trả lời của bạn đều được ẩn danh.

Khảo sát này cần khoảng **3 - 10 phút** để hoàn thành.

Về nghiên cứu

Less info

Mục đích của nghiên cứu này là gì?

Việc chia sẻ dữ liệu từ các nghiên cứu thử nghiệm lâm sàng càng ngày càng trở nên phổ biến. Chúng tôi cố gắng tìm hiểu xem các bộ dữ liệu được chia sẻ có được sử dụng lại hay không, những thách thức nào tồn tại trong việc truy cập và sử dụng lại dữ liệu cũng như tác động của việc sử dụng lại dữ liệu đối với nghiên cứu khoa học và sức khỏe cộng đồng nói chung.

Các lợi ích có thể từ nghiên cứu này là gì?

Dữ liệu từ nghiên cứu này sẽ có ích cho những người ra quyết định trong việc xác định tiêu chuẩn đo lường nào nên được ưu tiên để tăng cường việc sử dụng thứ cấp các dữ liệu. Chúng tôi dự đoán rằng việc gia tăng tái sử dụng các dữ liệu sẽ đưa đến kết quả cải thiện chất lượng và tính minh bạch trong khoa học, nâng cao sức khỏe cộng đồng và kết quả điều trị của bệnh nhân, đồng thời tái đầu tư hiệu quả vào nghiên cứu.

Đội ngũ nghiên cứu và thông tin

Nghiên cứu được dẫn dắt bởi các nhà nghiên cứu đến từ Đại học Oxford và Đơn vị Nghiên cứu Y học Nhiệt đới Oxford tại Mahidol với sự hợp tác của Anh Quốc, Kenya và Việt Nam. Nghiên cứu này được chấp thuận bởi Hội đồng Đạo đức trong Nghiên cứu Nhiệt đới Oxford (OxTREC), số tham chiếu:568-20.

Bảo vệ dữ liệu

Trong khi hoàn thành khảo sát, bạn sẽ phải cung cấp các thông tin về bản thân bạn (‘thông tin cá nhân’). Quy định về bảo mật dữ liệu yêu cầu rằng chúng tôi tuyên bố các cơ sở pháp lí trong xử lí các thông tin về bạn. Trong trường hợp của nghiên cứu, đây là “một nhiệm vụ vì lợi ích cộng đồng”. Trường Đại học Oxford là cơ quan kiểm soát dữ liệu và có trách nhiệm bảo vệ các thông tin của bạn và sử dụng theo Quy định chung về Bảo vệ dữ liệu và phù hợp với các luật bảo vệ dữ liệu liên quan. Thông tin của bạn sẽ được lưu giữ an toàn theo các chính sách và quy trình của trường đại học. Nhiều thông tin hơn có sẵn trên trang điện tử An toàn thông tin của trường website. Thông tin về các quyền liên quan đến dữ liệu cá nhân của bạn được giải thích tại đây. Các kết quả từ dự án nghiên cứu này sẽ được công bố thông qua các báo cáo nghiên cứu, các ấn phẩm/ bài báo và các bài thuyết trình. Chúng tôi sẽ xử lí dữ liệu của bạn cho các mục đích này chỉ khi bạn đã đồng ý cho chúng tôi thực hiện điều đó, bằng cách đánh dấu vào ô thích hợp. Các câu trả lời của bạn sẽ được ẩn

danh, do cuộc khảo sát không thu thập tên, địa chỉ email hoặc địa chỉ IP của bạn. Do đó, chúng tôi sẽ không thể rút lại câu trả lời của bạn sau khi bạn đã gửi đi câu trả lời. Nếu bạn thoát khỏi cuộc phỏng vấn trước khi gửi câu trả lời, các dữ liệu của bạn sẽ không được lưu trữ lại.

Liên hệ

Nếu bạn có bất cứ câu hỏi hay thắc mắc nào về việc sử dụng dữ liệu của bạn, vui lòng liên hệ chúng tôi tại reuse@tropmedres.ac hoặc số điện thoại +66 02 203 6333 Ext 8302 Nếu bạn muốn liên hệ với ai đó độc lập với đội ngũ nghiên cứu, bạn có thể gửi thư điện tử đến Hội đồng Đạo đức trong Nghiên cứu Nhiệt đới Oxford (OxTREC): oxtrec@admin.ox.ac.uk

○ Tôi đồng ý tham gia vào khảo sát. Tôi hiểu rằng sự tham gia của tôi là tự nguyện và tôi có thể rút lui vào bất cứ lúc nào.

Anh/chị đã từng sử dụng dữ liệu nghiên cứu lâm sàng do các nhà nghiên cứu khác chia sẻ chưa?

- ○ Nếu có, anh/chị vui lòng thực hiện Bản khảo sát Số 1
- ○ Nếu không, anh/chị vui lòng thực hiện Bản khảo sát Số 2

1) Anh/chị đã từng sử dụng loại dữ liệu nào do các nhà nghiên cứu khác chia sẻ? (chọn các ô phù hợp)

- ☐ Dữ liệu các thử nghiệm lâm sàng
- ☐ Các khảo sát cắt ngang về y tế
- ☐ Các bộ dữ liệu nghiên cứu quan sát đoàn hệ
- ☐ Các bộ dữ liệu giám sát
- ☐ Dữ liệu hành chính
- ☐ Sổ đăng ký bệnh nhân/bệnh
- ☐ Dược lý học
- ☐ Sinh học phân tử
- ☐ Họ omics (các bộ gen, phiên mã gen, nghiên cứu protein, các bộ gen biểu sinh, hệ chuyển hóa)
- ☐ Khoa học xã hội
- ☐ Kinh tế sức khỏe
- ☐ Các loại dữ liệu khác

Nếu bạn chọn Khác, vui lòng ghi rõ:

[ ]

2) Anh/chị đã truy cập vào các bộ dữ liệu trên bằng cách nào?

- ☐ Gửi yêu cầu truy cập cho người thu thập/quản lý dữ liệu
- ☐ Tải về từ các trang web/ kho dữ liệu công cộng
- ☐ Gửi yêu cầu truy cập thông qua một Hội đồng Truy cập Dữ liệu
- ☐ Những cách khác

Nếu bạn chọn Khác, vui lòng ghi rõ:

____

3) Anh/chị đã gửi yêu cầu truy cập dữ liệu trung bình bao nhiêu lần trong vòng 5 năm qua ? *Nếu hơn 5 lần*, thì 3a. Anh/chị đã yêu cầu truy cập cụ thể bao nhiêu lần trong từng 5 năm qua?

Trong năm 2017

Trong năm 2018

Trong năm 2019

Trong năm 2020

Trong năm 2021

4) Anh/chị sử dụng dữ liệu đó với mục đích gì?

- ☐ Lên kế hoạch và thiết kế các nghiên cứu mới (ví dụ: nhận định tính khả thi của nghiên cứu mới)
- ☐ Phân tích tổng hợp
- ☐ Thiết kế mô hình toán học
- ☐ Thử các thuật toán trí tuệ nhân tạo
- ☐ Tái phân tích để xác minh các phát hiện của nghiên cứu
- ☐ Phân tích thứ cấp (gồm đặt ra/kiểm nghiệm giả quyết mới)
- ☐ Dữ liệu cơ sở cho các nghiên cứu mới
- ☐ Giảng dạy
- ☐ Đánh giá tài liệu
- ☐ Dùng để đăng ký thuốc/thiết bị y tế
- ☐ Phát triển các bộ hướng dẫn/chính sách y tế sức khỏe
- ☐ Mục đích khác

Nếu bạn chọn Khác, vui lòng ghi rõ:

[ ]

5) Các đầu ra từ dữ liệu đó là gì? Chọn một hoặc nhiều các loại đầu ra phù hợp, và ghi rõ số lượng đầu ra cho từng loại đã chọn.

- ☐ Bài nghiên cứu đã xuất bản
- ☐ Bài thuyết trình
- ☐ Luận văn hoặc luận án tốt nghiệp
- ☐ Báo cáo
- ☐ Chương sách
- ☐ Các mô hình toán học đã kiểm chứng
- ☐ Phép thử các thuật toán trí tuệ nhân tạo
- ☐ Kiểm nghiệm các mã phầm mềm
- ☐ Bài viết trên blog
- ☐ Bài viết trên các kênh truyền thông xã hội
- ☐ Đầu ra khác
- ☐ Không có đầu ra nào từ việc sử dụng các dữ liệu này

Nếu bạn chọn Khác, vui lòng ghi rõ:

Tổng số bài báo nghiên cứu đã xuất bản

- ○ 1-4
- ○ 5-10
- ○ Nhiều hơn 10

Tổng số bài thuyết trình

- ○ 1-4
- ○ 5-10
- ○ Nhiều hơn 10

Tổng số luận văn hoặc luận án tốt nghiệp

- ○ 1-4
- ○ 5-10
- ○ Nhiều hơn 10

Tổng số báo cáo

- ○ 1-4
- ○ 5-10
- ○ More than 10

Tổng số chương sách

- ○ 1-4
- ○ 5-10
- ○ Nhiều hơn 10

Tổng số mô hình toán học đã được kiểm chứng

- 1-4
- 5-10
- Nhiều hơn 10

Tổng số phép thử các thuật toán trí tuệ nhân tạo

- 1-4
- 5-10
- Nhiều hơn 10

Tổng số lần kiểm nghiệm các mã phầm mềm

- 1-4
- 5-10
- Nhiều hơn 10

Tổng số bài đăng trên blog

- 1-4
- 5-10
- Nhiều hơn 10

Tổng số bài đăng trên các kênh mạng xã hội

- ○ 1-4
- ○ 5-10
- ○ Nhiều hơn 10

Cho biết tổng số sản phẩm đầu ra được đề cập trong phần ‘Khác’.

- ○ 1-4
- ○ 5-10
- ○ Nhiều hơn 10

6) Kết quả từ việc sử dụng dữ liệu được chia sẻ đó là gì?

- ☐ Kết quả từ nghiên cứu đã công bố được kiểm nghiệm/xác minh
- ☐ Các nghiên cứu hiện hành bị thu hồi lại
- ☐ Hoàn thành chương trình Thạc sỹ hoặc Tiến sĩ
- ☐ Tạo việc làm mới, ví dụ như tuyển thực tập sinh hoặc người phân tích dữ liệu
- ☐ Thăng tiến cho tôi/cộng sự của tôi
- ☐ Tăng các trích dẫn và khả năng hiển thị cho bản thân hoặc đơn vị tôi làm việc
- ☐ Các nghiên cứu có sử dụng dữ liệu thứ cấp làm cơ sở được triển khai
- ☐ Phân tích kết quả này có ảnh hưởng đến thiết kế của nghiên cứu mới
- ☐ Nhiều cơ hội hợp tác với các nhà nghiên cứu khác
- ☐ Được mời trình bày/phát biểu tại các diễn đàn thảo luận của các chuyên gia
- ☐ Được các cơ quan có thẩm quyền,ví dụ như cơ quan chính phủ hoặc WHO ghi nhận và đề cập
- ☐ Các phát hiện được đưa vào bộ hướng dẫn điều trị hoặc các chính sách về y tế
- ☐ Đăng ký thuốc hoặc thiết bị y tế mới
- ☐ Tài trợ cho nghiên cứu của tôi tăng lên
- ☐ Lợi ích tài chính từ việc đăng ký thuốc/thiết bị y tế mới
- ☐ Các lợi ích tài chính cá nhân, ví dụ như nhận được phí tư vấn cho phân tích thứ cấp
- ☐ Nghiên cứu của tôi hoặc đơn vị của tôi được tài trợ
- ☐ Các kết quả khác
- ☐ Không có kết quả nào

Nếu bạn chọn Khác, vui lòng ghi rõ:

[ ]

7) Anh/chị gặp phải thách thức gì khi tái sử dụng các dữ liệu nghiên cứu được thu thập từ trước

- ☐ Tìm kiếm dữ liệu liên quan đến công việc của tôi
- ☐ Truy cập dữ liệu
- ☐ Sử dụng dữ liệu
- ☐ Tôi không gặp phải thách thức gì
- ☐ Các thách thức khác

Nếu bạn chọn Khác, vui lòng ghi rõ:

8) Một số vấn đề thường gặp khi truy cập các bộ dữ liệu được liệt kê bên dưới. Dựa theo kinh nghiệm của anh/chị, các vấn đề này có ảnh hưởng như thế nào đến nghiên cứu/ dự án của anh/chị. Nếu anh/chị không gặp phải những vấn đền này, cũng vui lòng ghi nhận. *(0=Không ảnh hưởng; 1=Ít ảnh hưởng; 2=Có ảnh hưởng; 3=Ảnh hưởng nhiều; 4= Không gặp phải vấn đề này)*

	0	1	2	3	4
Thời gian thu thập dữ liệu kéo dài	☐	☐	☐	☐	☐
Dữ liệu không có sẵn tại thời điểm công bố các phát hiện của nghiên cứu	☐	☐	☐	☐	☐
Quy trình truy cập dữ liệu không rõ ràng	☐	☐	☐	☐	☐
Nhiều quy trình/thủ tục vất vả	☐	☐	☐	☐	☐
Dữ liệu không còn được lưu trữ ở kho dữ liệu	☐	☐	☐	☐	☐
Người cung cấp dữ liệu phản hồi chậm hoặc không phản hồi	☐	☐	☐	☐	☐
Những hạn chế về sự riêng tư, vấn đề đạo đức và pháp lý của dữ liệu	☐	☐	☐	☐	☐
Bị từ chối trao quyền truy cập	☐	☐	☐	☐	☐
Chi phí cho dữ liệu	☐	☐	☐	☐	☐
Dữ liệu được cung cấp bị hạn chế	☐	☐	☐	☐	☐

Các vấn đề khác

9) Một số khó khăn thường gặp khi *sử dụng* bộ dữ liệu được liệt kê bên dưới. Dựa theo kinh nghiệm của anh/chị, các khó khăn này có ảnh hưởng như thế nào đến nghiên cứu/ dự án của anh/chị. *(0=Không ảnh hưởng; 1=Ít ảnh hưởng; 2=Có ảnh hưởng; 3=Ảnh hưởng nhiều; 4= Không gặp phải vấn đề này)*

	0	1	2	3	4
Bộ dữ liệu không bao gồm các biến dữ liệu tôi cần	☐	☐	☐	☐	☐
Định dạng/cấu trúc dữ liệu không sử dụng được	☐	☐	☐	☐	☐
Dữ liệu lỗi hoặc không đồng nhất	☐	☐	☐	☐	☐
Bộ dữ liệu không hoàn thiện (thiếu nhiều giá trị)	☐	☐	☐	☐	☐
Khó khăn trong việc đọc hiểu dữ liệu (siêu dữ liệu không đầy đủ, ví dụ như không có từ điển dữ liệu)	☐	☐	☐	☐	☐
Thiết kế nghiên cứu không phù hợp	☐	☐	☐	☐	☐
Dữ liệu không đầy đủ, ví dụ như kích thước mẫu quá nhỏ	☐	☐	☐	☐	☐
Dữ liệu bằng một ngôn ngữ khác	☐	☐	☐	☐	☐
Siêu dữ liệu không hoàn thiện	☐	☐	☐	☐	☐
Thiếu nguồn lực để sử dụng dữ liệu, ví dụ như thiếu chuẩn bị, chú thích hoặc không trao đổi với các nhà nghiên cứu, thiếu công nghệ điện toán	☐	☐	☐	☐	☐

Các vấn đề khác

10) Anh/chị cần hỗ trợ/nguồn lực gì để có thể truy cập và sử dụng hiệu quả dữ liệu từ các nghiên cứu khác? *(1 =Ít hữu ích nhất 5 = hữu ích nhất)*

	1	2	3	4	5
Kho dữ liệu: nơi tìm các dữ liệu liên quan	☐	☐	☐	☐	☐
Cấp phép cho dữ liệu: điều khoản sử dụng dữ liệu	☐	☐	☐	☐	☐
Phân tích dữ liệu: phương pháp và công cụ thích hợp để tổng hợp và sử dụng dữ liệu	☐	☐	☐	☐	☐
Quy ước nghiên cứu: cách sử dụng dữ liệu có trách nhiệm (tác quyền, công nhận đóng góp, sở hữu trí tuệ)	☐	☐	☐	☐	☐
Hỗ trợ pháp lý: đàm phán và triển khai các thỏa thuận chia sẻ dữ liệu	☐	☐	☐	☐	☐
Hỗ trợ tài chính: trường hợp dữ liệu có tính phí	☐	☐	☐	☐	☐

Các hỗ trợ khác

Về bạn

11) Lĩnh vực nghiên cứu chính của anh/chị là gì?

Nếu bạn chọn Khác, vui lòng ghi rõ:

12) Anh/chị đang làm việc ở quốc gia nào? *(Ví dụ nếu anh/chị đang làm việc cho một trường đại học tại Thụy Sĩ và đang công tác thực địa ở Indonesia, vui lòng chọn Thụy Điển, là quốc gia nơi đơn vị của anh/chị hoạt động chính)*

13) Đơn vị của anh/chị là:

- ◯ Đại học hoặc đơn vị nghiên cứu học thuật
- ◯ Viện nghiên cứu công hoặc của chính phủ
- ◯ Tổ chức trên nền tảng tôn giáo hoặc phi chính phủ
- ◯ Tổ chức thương mại (ví dụ: công ty dược)
- ◯ Hội đồng Đạo đức
- ◯ Cơ quan pháp lý
- ◯ Quỹ nghiên cứu
- ◯ Đơn vị khác

Nếu bạn chọn Khác, vui lòng ghi rõ:

14) Chức vụ/ vai trò của anh chị là:

- ○ Nhà nghiên cứu lâm sàng
- ○ Nhà thống kê
- ○ Nhà dịch tễ học
- ○ Người quản lý dữ liệu
- ○ Nhà khoa học dữ liệu, ví dụ như chuyên gia trí tuệ nhân tạo, nhà tạo mô hình toán học
- ○ Nhà thông tin sinh học"
- ○ Nghiên cứu hỗ trợ nghề nghiệp ví dụ như Quản lý Dự án, Quản lý Nghiên cứu hoặc Thực hành Lâm sàng
- ○ Các chức vụ khác

Nếu bạn chọn Khác, vui lòng ghi rõ:

14a) Ở chức vụ đó, anh/chị là?

More info

- ○ Nghiên cứu viên cấp cao
- ○ Nghiên cứu viên nhiều năm kinh nghiệm
- ○ Nghiên cứu viên ít năm kinh nghiệm
- ○ Nghiên cứu sinh

15) Anh/chị thuộc nhóm tuổi nào?

16) Giới tính của anh/chị?

- Nam
- Nữ
- Giới tính khác
- Không muốn trả lời

17) Anh/chị có điều gì muốn chia sẻ gì với chúng tôi?

1) Vì sao anh/chị không sử dụng dữ liệu đã được thu thập từ các nhà nghiên cứu khác?

- ○ Tôi không cần sử dụng các dữ liệu khác cho công việc của tôi
- ○ Tôi không tìm được dữ liệu liên quan đến dự án của tôi
- ○ Tôi không truy cập được dữ liệu đó
- ○ Tôi gặp khó khăn trong việc sử dụng dữ liệu
- ○ Lý do khác

Nếu bạn chọn Khác, vui lòng ghi rõ:

1a) Một số vấn đề hay gặp phải khi truy cập các bộ dữ liệu chung được liệt kê bên dưới. Dựa trên kinh nghiệm của anh/chị, những vấn đề nào có ảnh hưởng đến dự án của các anh/chị. Nếu anh/chị không gặp phải những vấn đền này, cũng vui lòng ghi nhận. (0=Không ảnh hưởng; 1=Ít ảnh hưởng; 2=Có ảnh hưởng; 3=Ảnh hưởng nhiều; 4= Không gặp phải vấn đề này)

Please don't select more than 1 answer(s) per row.

Please select at least 1 answer(s).

	0	1	2	3	4
Thời gian thu thập dữ liệu kéo dài	☐	☐	☐	☐	☐
Dữ liệu không có sẵn tại thời điểm công bố các phát hiện của nghiên cứu	☐	☐	☐	☐	☐
Quy trình truy cập dữ liệu không rõ ràng	☐	☐	☐	☐	☐
Nhiều quy trình/thủ tục vất vả	☐	☐	☐	☐	☐
Dữ liệu không còn được lưu trữ ở kho dữ liệu	☐	☐	☐	☐	☐
Người cung cấp dữ liệu phản hồi chậm hoặc không phản hồi	☐	☐	☐	☐	☐
Những hạn chế về sự riêng tư, vấn đề đạo đức và pháp lý của dữ liệu	☐	☐	☐	☐	☐
Bị từ chối trao quyền truy cập	☐	☐	☐	☐	☐

Chi phí cho dữ liệu	☐	☐	☐	☐	☐
Dữ liệu được cung cấp bị hạn chế	☐	☐	☐	☐	☐

Các vấn đề khác

1a) Một số khó khăn thường gặp khi sử dụng bộ dữ liệu chung được liệt kê bên dưới. Dựa theo kinh nghiệm của anh/chị, các khó khăn này có ảnh hưởng như thế nào đến nghiên cứu/ dự án của anh/chị. *(0=Không ảnh hưởng; 1=Ít ảnh hưởng; 2=Có ảnh hưởng; 3=Ảnh hưởng nhiều; 4= Không gặp phải vấn đề này)*

Bộ dữ liệu không bao gồm các biến dữ liệu tôi cần	☐	☐	☐	☐	☐
Định dạng/cấu trúc dữ liệu không sử dụng được	☐	☐	☐	☐	☐
Dữ liệu lỗi hoặc không đồng nhất	☐	☐	☐	☐	☐
Bộ dữ liệu không hoàn thiện (thiếu nhiều giá trị)	☐	☐	☐	☐	☐
Khó khăn trong việc đọc hiểu dữ liệu (siêu dữ liệu không đầy đủ, ví dụ như không có từ điển dữ liệu)	☐	☐	☐	☐	☐
Thiết kế nghiên cứu không phù hợp	☐	☐	☐	☐	☐
Dữ liệu không đầy đủ, ví dụ như kích thước mẫu quá nhỏ	☐	☐	☐	☐	☐
Dữ liệu bằng một ngôn ngữ khác	☐	☐	☐	☐	☐
Siêu dữ liệu không hoàn thiện	☐	☐	☐	☐	☐
Thiếu nguồn lực để sử dụng dữ liệu, ví dụ như thiếu chuẩn bị, chú thích hoặc không trao đổi với các nhà nghiên cứu, thiếu công nghệ điện toán	☐	☐	☐	☐	☐

Các khó khăn khác

2) Những hỗ trợ/nguồn lực nào có thể giúp anh/chị truy cập và sử dụng dữ liệu từ các nghiên cứu viên khác một cách hiệu quả? *(1 =Ít hữu ích nhất 5 = hữu ích nhất)*

	1	2	3	4	5
Kho dữ liệu: nơi tìm các dữ liệu liên quan	☐	☐	☐	☐	☐
Cấp phép cho dữ liệu: điều khoản sử dụng dữ liệu	☐	☐	☐	☐	☐
Phân tích dữ liệu: phương pháp và công cụ thích hợp để tổng hợp và sử dụng dữ liệu	☐	☐	☐	☐	☐
Quy ước nghiên cứu: cách sử dụng dữ liệu có trách nhiệm (tác quyền, công nhận đóng góp, sở hữu trí tuệ)	☐	☐	☐	☐	☐
Hỗ trợ pháp lý: đàm phán và triển khai các thỏa thuận chia sẻ dữ liệu	☐	☐	☐	☐	☐
Hỗ trợ tài chính: trường hợp dữ liệu có tính phí	☐	☐	☐	☐	☐

Các hỗ trợ khác

Về bạn

3) Lĩnh vực nghiên cứu chính của anh/chị là gì?

If you selected Other, please specify:

4) Anh/chị đang làm việc ở quốc gia nào? *(Ví dụ nếu anh/chị đang làm việc cho một trường đại học tại Thụy Sĩ và đang công tác thực địa ở Indonesia, vui lòng chọn Thụy Điển, là quốc gia nơi đơn vị của anh/chị hoạt động chính)*

5) Đơn vị của anh/chị là :

- ○ Đại học hoặc đơn vị nghiên cứu học thuật
- ○ Viện nghiên cứu công hoặc của chính phủ
- ○ Tổ chức trên nền tảng tôn giáo hoặc phi chính phủ
- ○ Tổ chức thương mại (ví dụ: công ty dược)
- ○ Hội đồng Đạo đức
- ○ Cơ quan pháp lý
- ○ Quỹ nghiên cứu
- ○ Khác

Nếu bạn chọn Khác, vui lòng ghi rõ:

6) Hiện nay, anh/chị là?

- ○ Chuyên gia ngoài lĩnh vực học thuật
- ○ Nghiên cứu viên cấp cao
- ○ Nghiên cứu viên nhiều năm kinh nghiệm
- ○ Nghiên cứu viên ít năm kinh nghiệm
- ○ Nghiên cứu sinh
- ○ Khác

Nếu bạn chọn Khác, vui lòng ghi rõ:

7) Anh/chị thuộc nhóm tuổi nào?

8) Giới tính của anh/chị?

- ○ Nam
- ○ Nữ
- ○ Giới tính khác
- ○ Không muốn trả lời

9) Anh/chị có điều gì muốn chia sẻ gì với chúng tôi?

cảm ơn bạn đã hoàn thành bài khảo sát này

Nếu bạn có bất kỳ câu hỏi nào về dự án này, vui lòng gửi email tới ***reuse@tropmedres.ac***

Key for selection options

5 - 3) Anh/chị đã gửi yêu cầu truy cập dữ liệu trung bình bao nhiêu lần trong vòng 5 năm qua ? ***Nếu hơn 5 lần*****, thì 3a. Anh/chị đã yêu cầu truy cập cụ thể bao nhiêu lần trong từng 5 năm qua?**

1
2
3
4
5
Nhiều hơn 5

13 - 11) Lĩnh vực nghiên cứu chính của anh/chị là gì?

Bệnh truyền nhiễm
Y tế công/Sức khỏe toàn cầu
Khoa học thí nghiệm lâm sàng
Miễn dịch học lâm sàng
Vi sinh học lâm sàng
Dịch tễ học
Di truyền học phân tử
Ký sinh trùng học
Nha khoa
Da liễu học
Phụ khoa
Thần kinh học
Điều dưỡng

Mô học
Lĩnh vực nghiên cứu khác

14 - 12) Anh/chị đang làm việc ở quốc gia nào? ***(Ví dụ nếu anh/chị đang làm việc cho một trường đại học tại Thụy Sĩ và đang công tác thực địa ở Indonesia, vui lòng chọn Thụy Điển, là quốc gia nơi đơn vị của anh/chị hoạt động chính)***

Afghanistan
Akrotiri
Albania
Algérie
American Samoa
Andorra
Angola
Anguilla
Antarctica
Antigua và Barbuda
Argentina
Armenia
Aruba
Ashmore and Cartier Islands
Úc
Áo
Azerbaijan
Bahamas, The
Bahrain
Bangladesh
Barbados
Bassas da India
Belarus
Bỉ
Belize
Bénin

Bermuda
Bhutan
Bolivia
Bosna và Hercegovina
Botswana
Bouvet Island
Brazil
British Indian Ocean Territory
British Virgin Islands
Brunei
Bulgaria
Burkina Faso
Burma
Burundi
Campuchia
Cameroon
Canada
Cabo Verde
Cayman Islands
Cộng hòa Trung Phi
Tchad
Chile
Trung Quốc
Christmas Island
Clipperton Island
Cocos (Keeling) Islands
Colombia
Comoros
Congo, Democratic Republic of the
Cộng hòa Congo
Cook Islands
Coral Sea Islands
Costa Rica

Bờ Biển Ngà
Croatia
Cuba
Síp
Cộng hòa Séc
Đan Mạch
Dhekelia
Djibouti
Dominica
Cộng hòa Dominica
Ecuador
Ai Cập
El Salvador
Guinea Xích Đạo
Eritrea
Estonia
Ethiopia
Europa Island
Falkland Islands (Islas Malvinas)
Faroe Islands
Fiji
Phần Lan
Pháp
French Guiana
French Polynesia
French Southern and Antarctic Lands
Gabon
Gambia, The
Gaza Strip
Gruzia
Đức
Ghana
Gibraltar

Glorioso Islands
Hy Lạp
Greenland
Grenada
Guadeloupe
Guam
Guatemala
Guernsey
Guinée
Guiné-Bissau
Guyana
Haiti
Heard Island and McDonald Islands
Holy See (Vatican City)
Honduras
Hong Kong
Hungary
Ai-len
Iceland
Indonesia
Iran
Iraq
Ai-len
Isle of Man
Israel
Ý
Jamaica
Jan Mayen
Nhật Bản
Jersey
Jordan
Juan de Nova Island
Kazakhstan

Kenya
Kiribati
Korea, North
Korea, South
Kuwait
Kyrgyzstan
Lào
Latvia
Lebanon
Lesotho
Liberia
Libya
Liechtenstein
Litva
Luxembourg
Macau
Macedonia
Madagascar
Malawi
Malaysia
Maldives
Mali
Malta
Quần đảo Marshall
Martinique
Mauritanie
Mauritius
Mayotte
México
Micronesia, Federated States of
Moldova
Monaco
Mông Cổ

Montenegro
Montserrat
Maroc
Mozambique
Namibia
Nauru
Navassa Island
Nepal
Hà Lan
Netherlands Antilles
New Caledonia
New Zealand
Nicaragua
Niger
Nigeria
Niue
Norfolk Island
Northern Mariana Islands
Na Uy
Oman
Pakistan
Palau
Panama
Papua New Guinea
Paracel Islands
Paraguay
Peru
Philippines
Pitcairn Islands
Ba Lan
Bồ Đào Nha
Puerto Rico
Qatar

Reunion
Romania
Nga
Rwanda
Saint Helena
Saint Kitts và Nevis
Saint Lucia
Saint Pierre and Miquelon
Saint Vincent và Grenadines
Samoa
San Marino
São Tomé và Príncipe
Ả Rập Saudi
Sénégal
Serbia
Seychelles
Sierra Leone
Singapore
Slovakia
Slovenia
Quần đảo Solomon
Somalia
Nam Phi
South Georgia and the South Sandwich Islands
Spain
Spratly Islands
Sri Lanka
Sudan
Suriname
Svalbard
Swaziland
Thụy Điển
Thụy Sĩ

Syria
Taiwan
Tajikistan
Tanzania
Thái Lan
Timor-Leste
Togo
Tokelau
Tonga
Trinidad và Tobago
Tromelin Island
Tunisia
Thổ Nhĩ Kỳ
Turkmenistan
Turks and Caicos Islands
Tuvalu
Uganda
Ukraine
Ả Rập Thống nhất
United Kingdom
Chủng Quốc Hoa Kỳ
Uruguay
Uzbekistan
Vanuatu
Venezuela
Việt Nam
Virgin Islands
Wake Island
Wallis and Futuna
West Bank
Western Sahara
Yemen

Zambia
Zimbabwe

17 - 15) Anh/chị thuộc nhóm tuổi nào?

18-24
25-34
35-44
45-54
55-64
65-74
75 hoặc hơn
Không muốn trả lời

22 - 3) Lĩnh vực nghiên cứu chính của anh/chị là gì?

Bệnh truyền nhiễm
Y tế công/Sức khỏe toàn cầu
Khoa học thí nghiệm lâm sàng
Miễn dịch học lâm sàng
Vi sinh học lâm sàng
Dịch tễ học
Di truyền học phân tử
Ký sinh trùng học
Nha khoa
Da liễu học
Phụ khoa
Thần kinh học
Y tá
Mô học
Other

23 - 4) Anh/chị đang làm việc ở quốc gia nào? ***(Ví dụ nếu anh/chị đang làm việc cho một trường đại học tại Thụy Sĩ và đang công tác thực địa ở Indonesia, vui lòng chọn Thụy Điển, là quốc gia nơi đơn vị của anh/chị hoạt động chính)***

Afghanistan
Akrotiri
Albania
Algérie
American Samoa
Andorra
Angola
Anguilla
Antarctica
Antigua và Barbuda
Argentina
Armenia
Aruba
Ashmore and Cartier Islands
Úc
Áo
Azerbaijan
Bahamas, The
Bahrain
Bangladesh
Barbados
Bassas da India
Belarus
Bỉ
Belize
Bénin
Bermuda
Bhutan
Bolivia
Bosna và Hercegovina

Botswana
Bouvet Island
Brazil
British Indian Ocean Territory
British Virgin Islands
Brunei
Bulgaria
Burkina Faso
Burma
Burundi
Campuchia
Cameroon
Canada
Cabo Verde
Cayman Islands
Cộng hòa Trung Phi
Tchad
Chile
Trung Quốc
Christmas Island
Clipperton Island
Cocos (Keeling) Islands
Colombia
Comoros
Congo, Democratic Republic of the
Cộng hòa Congo
Cook Islands
Coral Sea Islands
Costa Rica
Bờ Biển Ngà
Croatia
Cuba
Síp

Cộng hòa Séc
Đan Mạch
Dhekelia
Djibouti
Dominica
Cộng hòa Dominica
Ecuador
Ai Cập
El Salvador
Guinea Xích Đạo
Eritrea
Estonia
Ethiopia
Europa Island
Falkland Islands (Islas Malvinas)
Faroe Islands
Fiji
Phần Lan
Pháp
French Guiana
French Polynesia
French Southern and Antarctic Lands
Gabon
Gambia, The
Gaza Strip
Gruzia
Đức
Ghana
Gibraltar
Glorioso Islands
Hy Lạp
Greenland
Grenada

Guadeloupe
Guam
Guatemala
Guernsey
Guinée
Guiné-Bissau
Guyana
Haiti
Heard Island and McDonald Islands
Holy See (Vatican City)
Honduras
Hong Kong
Hungary
Ai-len
Iceland
Indonesia
Iran
Iraq
Ai-len
Isle of Man
Israel
Ý
Jamaica
Jan Mayen
Nhật Bản
Jersey
Jordan
Juan de Nova Island
Kazakhstan
Kenya
Kiribati
Korea, North
Korea, South

Kuwait
Kyrgyzstan
Lào
Latvia
Lebanon
Lesotho
Liberia
Libya
Liechtenstein
Litva
Luxembourg
Macau
Macedonia
Madagascar
Malawi
Malaysia
Maldives
Mali
Malta
Quần đảo Marshall
Martinique
Mauritanie
Mauritius
Mayotte
México
Micronesia, Federated States of
Moldova
Monaco
Mông Cổ
Montenegro
Montserrat
Maroc
Mozambique

Namibia
Nauru
Navassa Island
Nepal
Hà Lan
Netherlands Antilles
New Caledonia
New Zealand
Nicaragua
Niger
Nigeria
Niue
Norfolk Island
Northern Mariana Islands
Na Uy
Oman
Pakistan
Palau
Panama
Papua New Guinea
Paracel Islands
Paraguay
Peru
Philippines
Pitcairn Islands
Ba Lan
Bồ Đào Nha
Puerto Rico
Qatar
Reunion
Romania
Nga
Rwanda

Saint Helena
Saint Kitts và Nevis
Saint Lucia
Saint Pierre and Miquelon
Saint Vincent và Grenadines
Samoa
San Marino
São Tomé và Príncipe
Ả Rập Saudi
Sénégal
Serbia
Seychelles
Sierra Leone
Singapore
Slovakia
Slovenia
Quần đảo Solomon
Somalia
Nam Phi
South Georgia and the South Sandwich Islands
Spain
Spratly Islands
Sri Lanka
Sudan
Suriname
Svalbard
Swaziland
Thụy Điển
Thụy Sĩ
Syria
Taiwan
Tajikistan
Tanzania

Thái Lan
Timor-Leste
Togo
Tokelau
Tonga
Trinidad và Tobago
Tromelin Island
Tunisia
Thổ Nhĩ Kỳ
Turkmenistan
Turks and Caicos Islands
Tuvalu
Uganda
Ukraine
Ả Rập Thống nhất
United Kingdom
Chủng Quốc Hoa Kỳ
Uruguay
Uzbekistan
Vanuatu
Venezuela
Việt Nam
Virgin Islands
Wake Island
Wallis and Futuna
West Bank
Western Sahara
Yemen
Zambia
Zimbabwe

26 - 7) Anh/chị thuộc nhóm tuổi nào?

18-24
25-34
35-44
45-54
55-64
65-74
75 hoặc hơn
Không muốn trả lời
