# Supplementary material for: Investigating the Secondary Use of Clinical Research Data: Protocol for a Mixed Methods Study
Source: JMIR Res Protoc. 2023 Mar 6;12:e44875. doi: 10.2196/44875 (PMC10028503; doi:10.2196/44875)
Supplement: Multimedia Appendix 6 [file resprot_v12i1e44875_app6.docx]

**REUSE Study: In-Depth interview Guide**

**Study title**: Understanding the extent and impact of secondary use of clinical research data with a focus on Low and Middle Income Countries: a cross sectional mixed methods study (REUSE)

**Target group:** Individuals working in clinical research or with clinical research data

**Forum:** Face to face or Microsoft Teams

**Aim:**  To understand:

- What outputs are obtained from reuse of clinical research datasets?
- What benefit has secondary use of clinical research datasets had for researchers and the general public?
- How has reuse of clinical research datasets influenced transparency and quality of research?
- What difficulties do users experience with access and reuse of clinical research data?

**Demographics:** *these details may be completed ahead of the interview*

1. Career stage
   - Professor/Associate Professor
   - Senior researcher
   - Postdoctoral researcher
   - Early career researcher
   - Graduate student
   - Other_________________________
2. Job title/ role
   - Statistician
   - Epidemiologist
   - Artificial Intelligence expert
   - Mathematical Modeler
   - Clinical researcher
   - Project Manager
   - Research Governance professional
   - Data Manager
   - Other________________________________
3. Institution
   - Academic research organization
   - Government or public research institution
   - Non-Governmental or Faith-Based Organisation
   - Commercial organization (*e.g. pharmaceutical company)*
   - Ethical Review Committee
   - Professional society
   - Regulatory Authority
   - Research Sponsor
   - Research Funder
   - Disease Advocacy Group
   - Other________________________________

1. Geographical location

**Drop down list of countries**

1. Primary research discipline

- Clinical laboratory sciences
- Clinical immunology
- Clinical microbiology
- Epidemiology
- Molecular genetics
- Parasitology
- Dental science
- Dermatology
- Gynecology
- Neurology
- Nursing
- Histology
- Other______________________

**Data use history**

1. What types of data do you routinely use?
   - Clinical trials data
   - Cross-sectional health surveys
   - Observational cohort datasets
   - Surveillance datasets
   - Administrative data
   - Patient / Disease registries
   - Pharmacology
   - Molecular biology
   - Omics *(Genomics, Transcriptomics, Proteomics, Epigenomics, Metabolomics)*
   - Other______________________
2. How do you access the datasets?
   - Personal request to data collector
   - Download from a public website/repository
   - Request through a Data Access Committee
   - Other
3. How many times have you requested access to data on average, in the last 5 years: |__|

1. What did you use the data for?

**For the dataset(s) that this user requested from the institution’s DAC or from a repository**

1. How did you intend to use the data you requested from this institution or repository?
2. What outputs such as publications, dissertations, mathematical models, did you obtain from reuse of the dataset(s)?

12(a) Data reuse can have benefits for the individual researcher such as career progression, financial benefit, increased visibility. What effect has reuse of this dataset had for you as a researcher?

12(b) It is thought that sharing and reuse of data promotes transparency and quality of research. What is your take on this notion, in the context of work you have done with this dataset?

12(c) To what extent has your work with this dataset contributed to improvement of public health?

1. For one to use data, they need to be aware of what data exists, where to find it, how to access it and eventually be able to access it. What obstacles did you encounter while finding and accessing the data?
2. Using data collected by other people may present some challenges. What difficulties did you experience while using this data?
3. Majority of clinical research datasets that are available are not optimally used. In your opinion, what can be done to increase reuse of clinical research data.
4. In future, in which areas do you need the most support in accessing and using other researchers’ data?
