# Supplementary material for: Investigating the Secondary Use of Clinical Research Data: Protocol for a Mixed Methods Study
Source: JMIR Res Protoc. 2023 Mar 6;12:e44875. doi: 10.2196/44875 (PMC10028503; doi:10.2196/44875)
Supplement: Multimedia Appendix 7 [file resprot_v12i1e44875_app7.docx]

**Participant Information Sheet**

**(In-depth Interviews)**

| **Study title** | Understanding the extent and impact of secondary use of clinical research data: a cross sectional mixed methods study (REUSE) |
| --- | --- |
| **Principal investigator** | Naomi Waithira |
| **Sponsor** | University of Oxford |

1. **What is this current research?**

It is increasingly becoming the norm to share data from clinical research studies. We seek to understand if and how shared datasets are reused, what challenges exist in accessing and reusing the data and what impact data reuse has had on scientific research and general public health. We will conduct an online survey involving at least 400 respondents and in-depth interviews with 20-40 participants who have previously accessed and used shared datasets.

1. **The reason that you have been invited is because:**

You have requested access to clinical research data from [*Insert institution/repository name*] or have used clinical research data for secondary purposes.

1. **What does it involve?**

During the interview, study details will be explained to you. You will be given time to consider the information and the opportunity to ask questions to help you decide whether to participate in the study. If you wish to participate, the study staff will ask you to provide verbal consent.

If you agree to take part in this study, we will ask you to share your views and experience on:

(a) What outputs you obtained from reuse of clinical research datasets.

(b) What benefit/effect the outputs had for you, your institution, other researchers and the general public.

(c) How your reuse of data has influenced transparency and quality of research.

(d) What difficulties you experienced with access and reuse of clinical research data.

The interview will last approximately 40-60 minutes.

1. **What will happen to my data?**

All information gathered will be treated confidentially. The interview will be audio recorded. Audio recordings will be transcribed and translated to English if necessary. Any information that could identify you for example mention of names, institutions or contact details will be removed from the transcripts (de-identification). Only de-identified data collected from this study will be used for analysis. Findings from this research project will be disseminated through research reports and publications and anonymised quotes from your interview may be used in these reports. De-identified data may be shared with other researchers in line with the MORU’s data sharing policy.

Contact details such as email address, name and telephone number you provide will be stored separately from the interview transcript and will not be linked to anything you say during the interview. Audio recordings of interviews will be kept in a secure and access restricted place on password servers at Mahidol Oxford Research Unit. The audio recordings will be discarded after analysis is completed to allow deletion of your record should you withdraw consent.

1. **Are there any disadvantages of taking part?**

We are not aware of any disadvantages associated with the study beyond the time required for the interviews. However, if there are any questions that you do not want to answer, they can be skipped and the interview can be stopped at any time.

1. **Are there any advantages of taking part?**

There is no direct benefit to you for your participation in this study. The advantage of taking part in this study is that you will help us to contribute to the generation of new knowledge on current data reuse practices and the impact of data sharing.

1. **What are the compensations to taking part in this interview?**

As the interview will be conducted online, compensation and travel cost will not be offered. If the interview is held face to face, you may be offered a snack during the meeting.

1. **Who will have access to the information you give?**

The information you give us will be kept confidentially. To ensure this, your personal information will be available only to the study staff on this study. Direct access to the information will also be granted to authorize representatives from the sponsor, Ethics Committees and regulatory authorities to ensure compliance with regulations.

1. **What will happen when you refuse to participate?**

Your participation in this study is completely voluntary. You have the right to withdraw from the study at any time. If you do not take part in this study, there will be no effects on your relationship with study staff both now and in the future.

If you decide to withdraw during the course of the interview, we will delete the audio recording of the interview and any information that you have provided.

1. **What if you have any question?**

If you have any question or concern, you can contact;

Naomi Waithira Email: [Naomi@tropmedres.ac](mailto:Naomi@tropmedres.ac) Tel: +66902485967

Keitcheya Chotthanawathit Email: [Keitcheya@hotmail.com](mailto:Keitcheya@hotmail.com) Tel: +66-8245-4662

If you have not been treated as specified in this information sheet or you wish to know your rights, or in case you require to file a formal complaint and wish to contact someone independent of the study team, you can email the Oxford Tropical Research Ethics Committee (OxTREC) by email: oxtrec@admin.ox.ac.uk.

1. **Data protection**

The University of Oxford is the data controller with respect to your personal data, and as such will determine how your personal data is used in the study.

Further information about your rights with respect to your personal data is available from <http://www.admin.ox.ac.uk/councilsec/compliance/gdpr/individualrights/>.

**CONSENT FORM**

| **Study title** | Understanding the extent and impact of secondary use of clinical research data: a cross sectional mixed methods study (REUSE) |
| --- | --- |
| **Principal investigator** | Naomi Waithira |
| **Sponsor** | University of Oxford |

**Researcher to seek and record informed oral consent, after participant has had sufficient time to think about whether they want to take part.**

Please check the boxes to record that the question has been asked by the researcher and that the participant has responded in the affirmative:

| 1. Do you confirm that you have read the information sheet dated 13 Nov 2020 (version 1.0) for this study? Have you had the opportunity to consider the information, ask questions and have these answered satisfactorily? | Yes  No |
| --- | --- |
| 1. Do you understand that your participation is voluntary and that you are free to withdraw at any time without giving any reason, without legal rights being affected? | Yes  No |
| 1. Do you understand that relevant sections of data collected during the study may be looked at by authorized representatives from the sponsor, Ethics Committees and regulatory authorities to ensure compliance with regulations. Do you give permission for these individuals to have access to your records? | Yes  No |
| 1. Do you agree to the interview being audio recorded? | Yes  No |
| 1. Do you agree to take part in this study? | Yes  No |
| Additional: |  |
| 1. Do you agree for your de-identified data including anonymized quotes to be shared with other researchers in future? | Yes  No |

Name of Participant ________________________

Name of Researcher taking consent Date Signature

________________________ ______________ _____________________

**1 copy for participant (e.g. emailed securely to participant); 1 copy for researcher file*
